# Highly efficient engineered waste eggshell-fly ash for cadmium removal from aqueous solution

**DOI:** 10.1038/s41598-022-13664-6

**Published:** 2022-06-11

**Authors:** Adina-Elena Segneanu, Catalin Nicolae Marin, Gabriela Vlase, Claudiu Cepan, Maria Mihailescu, Cornelia Muntean, Ioan Grozescu

**Affiliations:** 1 Institute for Advanced Environmental Research -West University of Timisoara (ICAM -WUT), Oituz nr. 4, Timisoara, Romania; 2 West University of Timisoara, 4 Blvd.V.Parvan, 300223 Timisoara, Romania; 3 University Politehnica Timisoara, 2 P-ta Victoriei, 300006 Timisoara, Romania

**Keywords:** Ecology, Hydrology, Materials science

## Abstract

Sustainable waste and water management are key components of the newest EU policy regarding the circular economy. Simple, performant and inexpensive water treatment methods based on reusing waste are prerequisites for human health, sustainable development and environmental remediation. The design of performant, cost-effective absorbents represents a topical issue in wastewater treatment. This study aimed to investigate the development of a newly engineered adsorbent by functionalizing two different types of waste (industrial and food) with magnetic nanoparticles as environmentally friendly, highly efficient, cheap material for cadmium removal from aqueous solutions. This nano-engineered adsorbent (EFM) derived from waste eggshell and fly ash was used to remove the cadmium from the aqueous solution. SEM analysis has demonstrated that magnetite nanoparticles were successfully loaded with each waste. In addition, was obtained a double functionalization of the eggshell particles with ash and magnetite particles. As a result of this, the EFM surface area substantially increased, as confirmed by BET. A comprehensive characterization *(*BET, FT-IR, SEM, XRD and TGA) was performed to study the properties of this newly engineered adsorbent. Batch experiments were conducted to investigate the influence of different reaction parameters: temperature, pH, contact time, dosage adsorbent, initial concentration. Results showed that cadmium adsorption reached equilibrium in 120 min., at pH 6.5, for 0.25 g of adsorbent. The maximum efficiency was 99.9%. The adsorption isotherms research displayed that the Cd^2+^ adsorption fitted on the Freundlich model indicated a multi-molecular layer adsorption process. In addition, the thermodynamic study (ΔG < 0, ΔH > 0; ΔS > 0) shows that cadmium adsorption is a spontaneous and endothermic process. The adsorbent kinetic study was described with the pseudo-second-order model indicating a chemisorption mechanism. Desorption results showed that the nano-engineered adsorbent (EFM) can be reused. These data confirmed the possibility to enrich relevant theoretical knowledge in the field of waste recovery for obtaining newly designed adsorbents, performant and inexpensive for wastewater remediation.

## Introduction

The water pollution corroborated with the water scarcity issue has become a major concern not only in Europe but also worldwide. It is well known that the largest water consumers are recorded in industry and agriculture. In the next years, the exponential growth of water consumption is forecast in these economic sectors which will harm the quality and freshwater reserves. In this respect, the global emerging economy imperatively imposes a new strategy for the complete transformation of the present quo linear economy to a completely new concept based on the preservation and regeneration of natural capital. The newest EU policy regarding the circular economy imposed a completely different strategic approach allowing the development of innovative and highly efficient methods and technology designed to ensure the achievement of the main European objectives: (1) economical and energetic security; (2) increased competitiveness; (3) sustainable resources and waste management^1–3^.

Sustainable water management will be a permanent challenge in particular, due to several factors, including an increasing trend of the global population, pollution, water resource depletion and last but not least the wide world increased food, bioenergy and clean water demand^1,2^.

Consequently, rapid and effective measures are needed to remove pollutants and decontaminate the pollutant source^1–3^.

There are several categories of contaminants in industrial wastewater: heavy metals, organic compounds (dyes, pharmaceuticals, surfactants, phenols, pesticides, hydrocarbons, halogenated compounds, etc.), suspended solids, others. Heavy metals (Cd, Cu, Hg, Pb, Ni, Zn, As) represent one of the most frequent and hazardous types of water pollutants due to their high toxicity for human health and the negative impact on bio-systems. Although heavy metal industrial tainted wastewater is a relatively common problem at present, it is imperative to treat it to avoid extremely serious long-term ecological problems^4–8^.

Cadmium pollution must be considered a top priority and main public health concern. This transitional metal accumulates in the ecosystem and the human body, having a long half-life of about 25–30 years, is included in the category of teratogenic and carcinogenic substances by the World Health Organization^9–12^.

According to WHO recommendations, the maximum allowable concentration of cadmium in water is 3 μg/L^13,14^. Contamination of the environment with cadmium, food and beverages is the result of various anthropological activities, agriculture (phosphate fertilizers, pesticides), industry (mining, textile industry, metallurgical industry, electroplating, incineration of solid and liquid fuels, welding, batteries, and so on), improper storage or burning of waste high in cadmium, smoking, etc.^10–12,15,16^.

It is estimated that globally the amount of cadmium emissions is about 7000 t/year. Therefore, is necessary to adopt efficient ecological regulations and strategies regarding the limitation of pollution with this heavy metal. It should also be mentioned that, as a result of the measures imposed within the European Union, cadmium emissions have decreased by over 30%. However, minimizing heavy metal emissions remains a global priority^17–20^. Moreover, since the end of August 2021, maximum limits have been imposed in the EU on a range of foods, a measure that also aims to reduce food contamination by lead and cadmium^21^.

Conventional technologies used for the removal of heavy metals from industrial wastewater are quite various and are based on chemical, physical or combined processes (chemical precipitation, membrane separation, reverse osmosis, electrodialysis, extraction with different solvents, flocculation and adsorption)^2–6,15,22,23^. Their applicability on an industrial scale is limited by several factors: the efficiency of cadmium removal, cost-effectiveness, treatment time, simplicity, the generation of secondary chemicals (wastes), ecological aspects (possibility of heavy metal recycling), others^15,22^.

Adsorption is considered to be the most efficient and inexpensive method for removing heavy metals^15,16,22^. There are various studies on different categories of adsorbents use to remove cadmium from wastewater: natural and synthetic zeolites, natural clays, alumina, silica, polymer materials, activated carbon, chitosan, ferric oxides, etc.^22,24^.

A sustainable economy aims to increase the process performance at minimal costs. The raw material costs must play a key role in the development of any future technology.

Conversion of wastes as a source of raw materials for innovative and highly efficient materials for removing contaminants from wastewater from manufacturing processes represent an interesting challenge for researchers.

Magnetic nanoparticles, in addition to multiple other applications (drug delivery, sensors, chemotherapy, data storage, ferrofluids, nanotechnology, etc.), can be used in wastewater remediation as effective adsorbents for the removal of pollutants (heavy metals, phosphorus) due to their high specific area and stability, low-cost. An additional advantage is that after adsorption they can be easily recovered with a magnetic field^23,25,26^.

In the context of the current European circular economy action plan (CEAP) are required to identify efficient strategies based on extremely high-performance, versatile and cheap materials. Particular attention is paid to the valorization of waste in the development of advanced methods to increase the efficiency of resources and reduce the negative impact on water quality, soil productivity and food security^27^.

Recent studies have focused on the reuse of different agricultural and industrial wastes (sawdust, nuts shell, animal bones, crustacean shell, tea leaves, various fruits seed, vegetable or fruits peel, corncob, hen eggshell, coal ash and so on) for the adsorption of cadmium from aqueous solutions^15,16,22,28–31^.

Studies focused on the possibilities of reusing the fly ash (waste from the power plants) have demonstrated its applicability in construction (for the preparation of cement, concrete) but also in the remediation of the environment, where it showed good adsorbent properties against various heavy metals or organic pollutants (phenols, dyes) from wastewater^16,22,32^.

Furthermore, fly ash is an environmentally friendly source of silica (SiO_2_) and alumina (Al_2_O_3_) (its major components), very cheap, available in large quantities^32^.

Eggshell waste has been classified by the Environmental Protection Agency as one of the main environmental pollutants generated by the food industry, with potential health hazards for humans^29^.

Globally, and especially in Asia, there is a steady increase in the amount of eggshell waste, which is why it is necessary to develop sustainable strategies to minimize the negative impact on the environment by reusing them as value-added raw material sources^29,31^.

Research in this regard has shown that, due to its very high content (about 95%) of calcium carbonate, it is a much more affordable substitute than activated carbon for immobilizing heavy metals in wastewater^15,30,33^.

Studies on the adsorption performance of heavy metals have been shown to achieve a very high yield (93%) at 25 °C, short contact time (1.5 h) and a neutral pH. Moreover, the cost-effective method can be improved by reducing the size of the eggshell particle to nanometer size^29^.

The performance of an adsorbent is influenced by its surface and pore size. Engineering adsorbents have allowed optimizing the adsorption capacity, stability and selectivity^34^.

The functionalization of some wastes with magnetic iron nanoparticles ensures, in addition, the easy separation and recovery of adsorbent into an external magnetic field^35^.

Recent research has reported that α-FeOOH modified eggshells can improve the adsorbent properties of eggshell^29,36^.

Lately, the development of eggshell coated magnetic nanoparticles has demonstrated an efficiency of over 94% in removing cadmium from water^29,35^.

In this study, a new nano-engineered low-cost adsorbent from two different types of waste (eggshell and fly ash) were prepared for immobilizing cadmium from aqueous solutions. The mechanism of functionalization was the simultaneous loading of each waste (eggshell and fly ash) with magnetite nanoparticles.

To our best knowledge, this study reported first the possibility of concomitant reuse of industrial waste (fly ash) and food waste (eggshell) to remove cadmium from wastewater.

The materials used for the engineered adsorbent preparation are cheap, available in large quantities, environmentally friendly, have good performance in removing cadmium from aqueous solutions and are reusable. The new nano-engineered adsorbent obtained by us exhibits enhanced adsorbent properties of the waste and has magnetic properties that allow magnetic separation. The characteristics of this new low cost-adsorbent (adsorption isotherm model, thermodynamic studies, adsorption kinetics, adsorption mechanism, desorption kinetics, influence of various reaction parameters such as temperature, initial concentration, adsorbent dose, contact time, pH) were systematically investigated. In addition, the effect of different molar ratios between the two wastes on cadmium removal performance was also determined. The physico-chemical properties of this new adsorbent were studied in-depth through the joint analysis methods of Brunauer–Emmett–Teller (BET) surface area measurement, Fourier-transform infrared (FTIR) spectroscopy, scanning electron microscopy (SEM), thermogravimetric, analysis (TGA) and X-ray diffraction (XRD).

These data provide a new type of highly efficient, reusable and selective, low-cost adsorbent for the potential application in heavy metal wastewater remediation.

## Materials and methos

All used reagents and solvents were analytical grade and were acquired from commercial sources (Merck, WWR, Sigma-Aldrich) and used without further purification.

Electro-filter fly ash (with particle average size of 25–55 nm) was provided by Colterm Cogeneration Power station (Timisoara, Romania). The fly ash chemical composition was characterized using SEM/EDAX analysis before the preparation of absorbent.

Eggshells (ES) were collected from the household, washed five times with distillate water to remove any impurities. After that was dried in an oven at 50 °C for 24 h. Finally, the eggshells were crushed and sieved to obtain a powder with the size of particles between 125 and 250 μm^15^.

Industrial magnetite (with particle size of 50 nm) was commercially acquired from Jalutex, Romania. The magnetite was washed several times with distillate water to remove dust or any other impurities. Then were filtered and dried at 180 °C for 18 h. Finally, the dried magnetite cooled to room temperature was stored in a desiccator until it was used.

*Scanning electron microscope (SEM) micrographs* were conducted with SEM–EDS system (QUANTA INSPECT F50) equipped with field emission gun (FEG), 1.2 nm resolution and energy dispersive X-ray spectrometer (EDS) with MnK resolution of 133 eV.

*X-ray powder diffraction (XRD) pattern* was performed using Rigaku Ultima IV diffractometer equipped with a D/teX Ultra detector and operating at 40 kV and 40 mA with monochromatic CuKα radiation (λ = 1.5406 Å), in the 2θ range 10–80°, with a scan speed of 5°/min and a step of 0.01°. The XRD patterns were compared with those from ICDD Powder Diffraction Database, (ICDD file 04-015-9120). Frequency dependence was conducted with an Agilent LCR-meter (E-4980A type), at room temperature, over the frequency range (1 kHz to 2 MHz) and various values of the polarizing field. The duration of the measurement into a constant magnetic field, over the entire frequency range, was about 40 s. The average crystallite size and the phase content was calculated using the whole pattern profile fitting method (WPPF).

The *thermal analysis* was performed using a Perkin-Elmer Diamond TG/DTA/DTG (Perkin–Elmer, Waltham, MA, USA), in a synthetic air atmosphere (Linde-Gas). The analyses were performed in open aluminum crucibles, using a heating rate of 10 °C/min to 500 °C. Fourier transform infrared spectrum (FTIR) spectra were obtained by using KBr pellet method ranging from 4000 to 400 cm^−1^ with Perkin–Elmer Spectrum 100 FT-IR (Perkin–Elmer, Waltham, MA, USA).

*Brunauer–Emmett–Teller (BET)* analysis was conducted by using Nova 1200 e high-speed surface area and porosity analyzer (Quantachrome, Boynton Beach, FL, USA). Nitrogen adsorption/desorption isotherms were recorded at 77 K. The specific surface area was calculated by Brunauer–Emmett–Teller (BET) theory^37^. The surface areas and the *pore* size *distributions* were *calculated* by the BET (from multi-point regression in the 0.08–0.3 relative pressure range) and (Barrett-Joyner-Halenda) (*BJH) method*, respectively. The total pore volume was determined from the last point of the isotherm with a value close to 1^38^.

*The atomic absorption spectrophotometry* was performed using an atomic absorption spectrophotometer (Varian SpectrAA 280 FS adsorption, Varian, Palo Alto, CA, USA).

### Preparation of adsorbent

A newly nano-engineered adsorbent (EFM) was prepared from magnetite, eggshell and fly ash using two distinct molar ratios: 1:3:1 (magnetite:eggshell:fly ash) for **M1**, and respectively 1:1:3 (magnetite:eggshell:fly ash) for **M2**. And each of the obtained mixtures was subject to mechanical alloying in the mill with high energy balls at room temperature (22.5 °C), for 15 min.

### Single factor static adsorption experiment

Batch adsorption experiments using a single factor static adsorption method were conducted to investigate the performance of the prepared adsorbent to remove cadmium from water considering the influences of the main reaction parameters: initial concentration (1–33.5 mg/L), contact time (0–600 min), pH (3–7), adsorbent dosage (0.05–0.35 g) and temperature (5–50 °C). The adsorption experiment proceeded in a 100 mL conical flask containing 0.25 g absorbent and 50 mL of the metal ion stock solution (28.5 mg/L), pH 6.5. The resulting suspended solutions were placed into a thermostat shaker at (22.5 °C), 140 rpm for 120 min. After adsorption reached equilibrium, the mixed liquid was centrifuged, decanted and filtered (Φ185 mm filter paper). The initial and residual cadmium concentration was analysed by atomic absorption spectrophotometer. The residual heavy metal concentrations from all samples were detected by atomic absorption spectrophotometry. In this study, stock cadmium solution with 28.5 mg/L is prepared for the adsorption tests by dissolving the chemically pure cadmium nitrate in an appropriate amount of distilled water.

All standard heavy metal solutions with the selected concentration used in adsorption experiments were prepared by diluting the stock cadmium solution (28.5 mg/L) with distilled water. Standard 1 N HNO_3_ and 1 N NaOH solutions were used for pH adjustment. In order to obtain reproducible experimental results, all adsorption experiments were carried out in triplicate and averaged, and the obtained data and the result is accurate to 0.02%.

Statistically significant differences between the adsorbents used in this study: M1 and M2 were conducted using one-way analysis of variance (ANOVA) without replication was further used to test the null hypothesis of no significant differences in the applicability of M1 and M2 towards the cadmium adsorption^39^.

### Cadmium adsorption capacity and removal efficiency

The removal efficiency as well as the adsorption capacity were calculated based on the changes of cadmium concentration before and after the adsorption were calculated on the basis of the following Eqs. (1), (2):

1 $${\text{R}}\left( \% \right) = 1 - \frac{Ce}{{C_{0} }}100\%$$

2 $${\text{Q}} = \frac{{\left( {C0 - Ce} \right)}}{m}{\text{V}}\left( {{\text{mg}}\;{\text{Cd/g}}} \right)$$

where R (%) represent the removal efficiency; Q (mg Cd/g) is the adsorption capacity; V (mL) represent the working solution volume; C_0_ and C_e_ (mg/L) are cadmium initial concentration and respectively residual cadmium concentration in when the adsorption becomes equilibrium; m (g) is the adsorbent amount.

### Adsorption isotherms

The adsorption isotherm study was conducted for newly adsorbent material (0.25 g) with different initial heavy metal concentrations (1–33.5 mg/L) at 22.5 °C, pH 6.5 and the adsorption time was 2 h.

The Langmuir and Freundlich isotherms are expressed in Eqs. (3) and (4)

3 $${\text{Q}} = \frac{Qm - KL Ce}{{1 + KLCe}}$$

4 $${\text{Q}}_{{\text{e}}} = {\text{K}}_{{\text{F}}} Ce^{1/n}$$

The Langmuir model was used for determination of separation factor, R_L_, according to the next equation, Eq. (5).

5 $${\text{R}}_{{\text{L}}} = \frac{1}{{1 + {\text{K}}_{{\text{L}}} Q_{m} }}$$

where Q (mg/g) represent the equilibrium adsorption capacity; C_e_ (mg/L) = cadmium concentration in the solution at equilibrium. Q_m_ = theoretical maximum adsorption capacity; K_L_ (L/mg)—adsorption constant in Langmuir isotherm; K_F_ = the constant of Freundlich isotherm; n = heterogeneity factor of Freundlich isotherm.

### Effect of contact time

The effect of contact time on the adsorption process was investigated both for M1 and M2 following the next procedure: in five different conical flasks (100 mL) 0.25 g absorbent was weighted and added a constant volume (50 mL) of aqueous solution (with concentration of 28.5 mg/L cadmium) at pH 6.5 The flasks were kept at room temperature (22.5 °C) and 180 rpm, and small samples were collected at different times (0–600 min).

### Effect of pH value on absorption process

Adsorption experiments of cadmium on M1 and respectively M2 were performed at different pH values (varied from 3.0, 3.5, 4.0, 4.5, 5.0, 5.5, 6.0, 6.5 to 7.0), because higher pH values can result in precipitation of cadmium^15^.

Batch experiments were conducted in 100 mL conical flasks solution were added 0.25 g absorbent, a constant volume (50 mL) of cadmium solution (28.5 mg/L). The flasks were kept 120 min at room temperature (22.5 °C) and 180 rpm.

### Effect of adsorbent dosage

Sets of batch experiments were conducted to evaluate the effect of adsorbent dosage. For each set of analysis, seven different samples (S_1_ = 0.05 g; S_2_ = 0.10 g: S_3_ = 0.15 g; S_4_ = 0.20 g; S_5_ = 0.25 g; S_6_ = 0.30 g and respectively S_7_ = 0.35 g) from adsorbent material (both M1 and M2) were weighed. To each adsorbent sample (S_1_–S_7_) a constant volume of 50 mL synthetic water (cadmium solution) with concentration of 28.5 mg/L was added. The mixture (pH 6.5) was stirred at 180 rpm at room temperature for 2 h.

### Effect of initial concentration on cadmium removal efficiency

Set batch experiments were conducted both for M1 and M2 following the next procedure in conical flasks (100 mL) 0.25 g absorbent was weighted and added a constant volume (50 mL) of synthetic water with different values of cadmium concentration in the range 1.0 mgL–33.5 mg/L. The flasks were kept at room temperature (22.5 °C) for 120 min. and 180 rpm, and small samples were collected at different times (0–600 min).

### Effect of temperature on absorption process

Batch experiments were conducted to evaluate the effect of the temperatures on cadmium removal efficiency following the next procedure: in six different conical flasks (100 mL) 0.25 g absorbent was weighted and added a constant volume (50 mL) of synthetic water (with concentration of 28.5 mg/L cadmium) at pH 6.5 (adjusted with 1 N HNO_3_ or 1 N NaOH solution) and were stirred at 5 °C, 10 °C, 25 °C, 35 °C, 40 °C and 50 °C. Small sample of filtrate were collected.

### Comparison between the effect of contact time on adsorption process on raw materials and prepared adsorbent

The effect of contact time on the adsorption process of each raw material and of EFM adsorbent (M1 and M2) was investigated following the next procedure: in five different conical flasks (100 mL) 0.25 g absorbent was weighted and added a constant volume (50 mL) of aqueous solution (with concentration of 28.5 mg/L cadmium) at pH 6.5 (adjusted with 1 N HNO_3_ or 1 N NaOH solution). The flasks were kept at room temperature (22.5 °C) and 180 rpm, and small samples were collected at different times (0–720 min), filtered using Whatman filter papers (0.45 μm) and cadmium concentration measured using atomic absorption spectrophotometry.

### Adsorption kinetic model

Both adsorption reaction models and adsorption diffusion models were used to describe the kinetics of the heavy metal adsorption process on the prepared adsorbent. Thus, the pseudo-first-order kinetic model, pseudo-second-order kinetic model and intraparticle diffusion model were selected and fitted with the experimental data.

The pseudo-first-order kinetic model (Lagergren equation) is expressed in Eq. (6)

6 $${\text{log}}(q_{e} - q_{t} ) = \log q_{e} - \frac{{K_{1} t}}{2,303}$$

where qe (mg/g) = adsorption capacities at equilibrium. qt (mg/g) = adsorption capacities at the time t. K_1_ (min^−1^) = rate constant of adsorption kinetics.

The quasi-second-order kinetic model (Ho equation) is presented in the following Eq. (7):

7 $$\frac{1}{{q_{t} }} = \frac{1}{{K_{2 } q_{e}^{2} }} + \frac{1}{qe}$$

Herein K_2_ [mg/(g min)] = the constant of speed of pseudo second order.

The intraparticle diffusion model is described in the Eq. (8)

8 $$q_{t} = K_{i} { }t^{1/2} + {\text{ C}}$$

where Ki [mg/(g min^-1/2^)] = the intraparticle diffusion rate constant, qt (mg/g) = the amount of metal ions adsorbed at time t, C (mg/g) = constant related to the thickness of the boundary layer.

The adsorption kinetics study was conducted at constant temperature (25 °C) and pH value (pH = 6.5), 0.25 g absorbent and 25 mL cadmium solution. The samples were taken out at different times (0–250 min).

### Thermodynamic parameters of adsorption process

Evaluation of the prepared material thermodynamics properties (entropy (ΔS), enthalpy (ΔH), and Gibbs free energy (ΔG°) were determinate using the next equations (Eqs. 9–11):

9 $${\text{K}} = \frac{{\text{Q}}}{{{\text{C}}_{{\text{e}}} }}$$

10 $$\Delta {\text{G}} = - {\text{RT lnK}}$$

11 $$\Delta {\text{G}} = \Delta {\text{H}} - {\text{T}}\Delta {\text{S}}$$

Equation van't Hoff (Eq. 9), describe the correlation between enthalpy (ΔH) and entropy (ΔS)

12 $$\ln {\text{K = }}\frac{{\Delta {\text{S}}}}{{{\text{RT}}}} - \frac{{\Delta {\text{H}}}}{{{\text{RT}}}}$$

Herein, Q (mg/g) = adsorption capacity at equilibrium; *C*_e_ (mg/mL) = equilibrium adsorption concentration; K (mL/g) = adsorption equilibrium constant, ΔG (kJ/mol) represent the free energy variation value of the adsorption process or the Gibbs free energy; ΔH (kJ/mol) = enthalpy change value of adsorption process; ΔS [J/(mol K)] = entropy variable value; *R* = 8.314 J/(mol K) = the gas constant; T(K) = the absolute temperature.

The thermodynamic study was conducted for six different temperatures (278.15 K; 283.15 K; 298.15 K; 308.15 K; 313.15 K and 323.15 K), constant pH value (pH = 6.5), 0.25 g absorbent and 25 mL cadmium solution.

The adsorption thermodynamic diagram was plotted as lnK (abscissa) against 1/T (ordinate). The correlation coefficient R^2^ = 0.9757 demonstrates a good linear relationship between the two selected coordinates. Function: Y = − 0.0431X + 0.0587.

### Desorption study

Batch desorption experiments were conducted in 100 mL conical flasks solution were added 0.25 g absorbent, a constant volume (50 mL) of cadmium solution (28.5 mg/L), at constant temperature (25 °C) A volume of 10 mL of nitric acid (1.0 mol/L) or hydrochloric (1.0 mol/L) or NaOH (1.0 mol/L) was added, respectively. The flasks were kept at room temperature (22.5 °C) and 180 rpm and small samples were collected at different times (0–720 min), filtered using Whatman filter papers (0.45 μm) and residual cadmium concentration measured using atomic absorption spectrophotometry. The desorption rate was calculated from the next formula:

13 $$desorption \left( \% \right) = \frac{{q_{d} }}{{q_{a} }} 100$$

Herein, q_d_ = amount of metal ion desorbed, q_a_ = amount of metal ion adsorbed.

### The evaluation of the adsorbent regeneration

Batch experimental analysis was performed using the following procedures: conical flasks (100 mL) 0.25 g absorbent was weighted and added a constant volume (50 mL) of aqueous solution (with concentration of 28.5 mg/L cadmium) at room temperature (22.5 °C) and pH = 6.5. A volume of 25 ml of HNO_3_ 0.1 M was added. The mixture was stirred at 180 rpm at room temperature for 5 h and then filtered (0.45 μm). The heavy metal residual concentration was analysed using atomic absorption spectroscopy.

## Results and discussion

### Characterization of EFM adsorbent

#### BET analysis

The surface properties of newly prepared adsorbent and its components were investigated through nitrogen adsorption–desorption isotherms. Table 1 presents the results of the adsorbent the textural properties assessment.

**Table 1:** EFM adsorbent and raw materials used (magnetite, eggshell and fly ash)—specific surface area determinate by Brunauer–Emmett–Teller theory (BET).

Sample	Surface area (m^2^/g)	Average pore size diameter (nm)	Total pore volume (cm^3^/g)
Eggshell	0.67	24.534	0.011
Fly ash	4.961	8.993	0.020
Magnetite	17.8	3.7	0.044
M1	25.196	1.803	0.0003
M2	26.866	1.101	0.00011

From the quantitative data reported in Table 1 it can be observed that eggshell BET/N_2_ surface area is 0.67 m^2^/g, value similar to that in the literature^15,31,40^.

According to Table 1 the BET/N_2_ specific surface area for fly ash, is 4.961 m^2^/g. Apparently the value obtained in this study seems higher than in the data reported in the literature (0.414 m^2^/g). However, it should be noted that both the type of ash used and the experimental conditions of this study differ from the data reported in the literature^16,22^.

As expected, very different values were obtained for the specific surface of M1 (25.196 m^2^/g) and respectively 26.866 m^2^/g for M2, which correspond to the adsorbent prepared in the two molar ratios studied. This difference can be justified by the variation of the ratio between eggshell and fly ash in M1 and M2, respectively.

**Figure 1:**
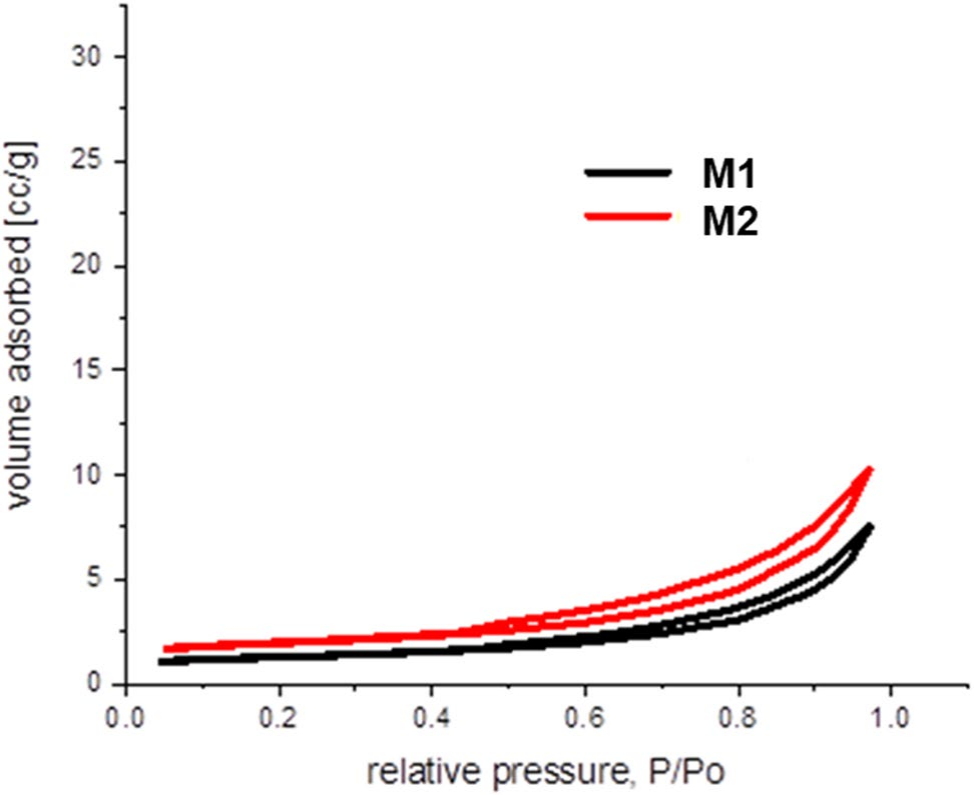
The nitrogen adsorption–desorption isotherms for EFM adsorbent.

Physical properties of adsorbent (pore size, pore volume) were investigated using the low-temperature (77 K) nitrogen adsorption–desorption isotherms. As presented in Fig. 1 the isotherms of EFM fitted in a type II isotherm with a H3 hysteresis loop, indicating a macroporous structure for the both adsorbent molar ratio (M1 and M2)^38^.

#### XRD studies

The mineralogical compositions of the raw materials as well as of the adsorbent were studied through XRD analysis. The size of the crystalline domains was evaluated by means of the Debye–Scherrer formula (Eq. 14)^41^,

14 $$D = \frac{0.89\lambda }{{\beta \cos \left( \theta \right)}}$$

where $\lambda$ is the X-ray wavelength of Cu K-α ($\lambda = 0.15406\;{\text{nm}}$), $\beta$ is the full width at half maximum in radians and $\theta$ is the Bragg angle.

From the most prominent peak, one gets D = 21.6 nm.

**Figure 2:**
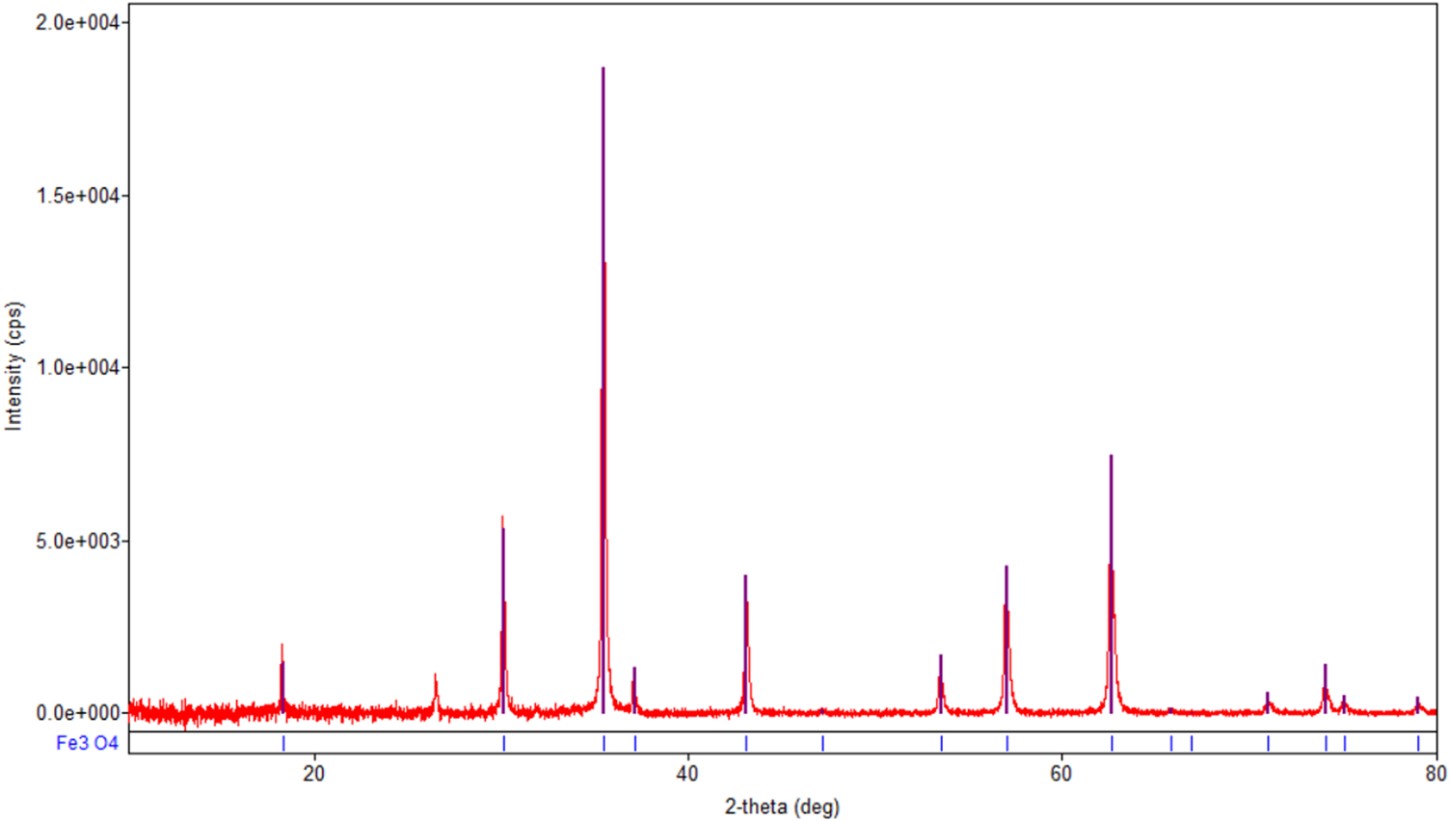
XRD spectra of magnetite.

**Figure 3:**
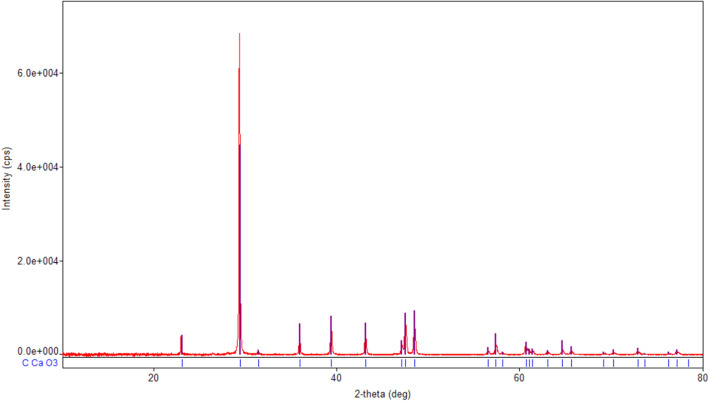
XRD spectra of eggshell.

The XRD spectrum of magnetite sample (Fig. 2) shows the diffraction peaks of the well crystallized spinel phase magnetite Fe_3_O_4_ (COD 9005837) with average crystallite size of 21.6 nm. In the XRD spectrum of eggshell sample (Fig. 3) are recorded the diffraction peaks of the single phase well crystallized calcite CaCO_3_ (COD 9000965) with mean crystallite size of 125.8 nm^15,40^.

**Figure 4:**
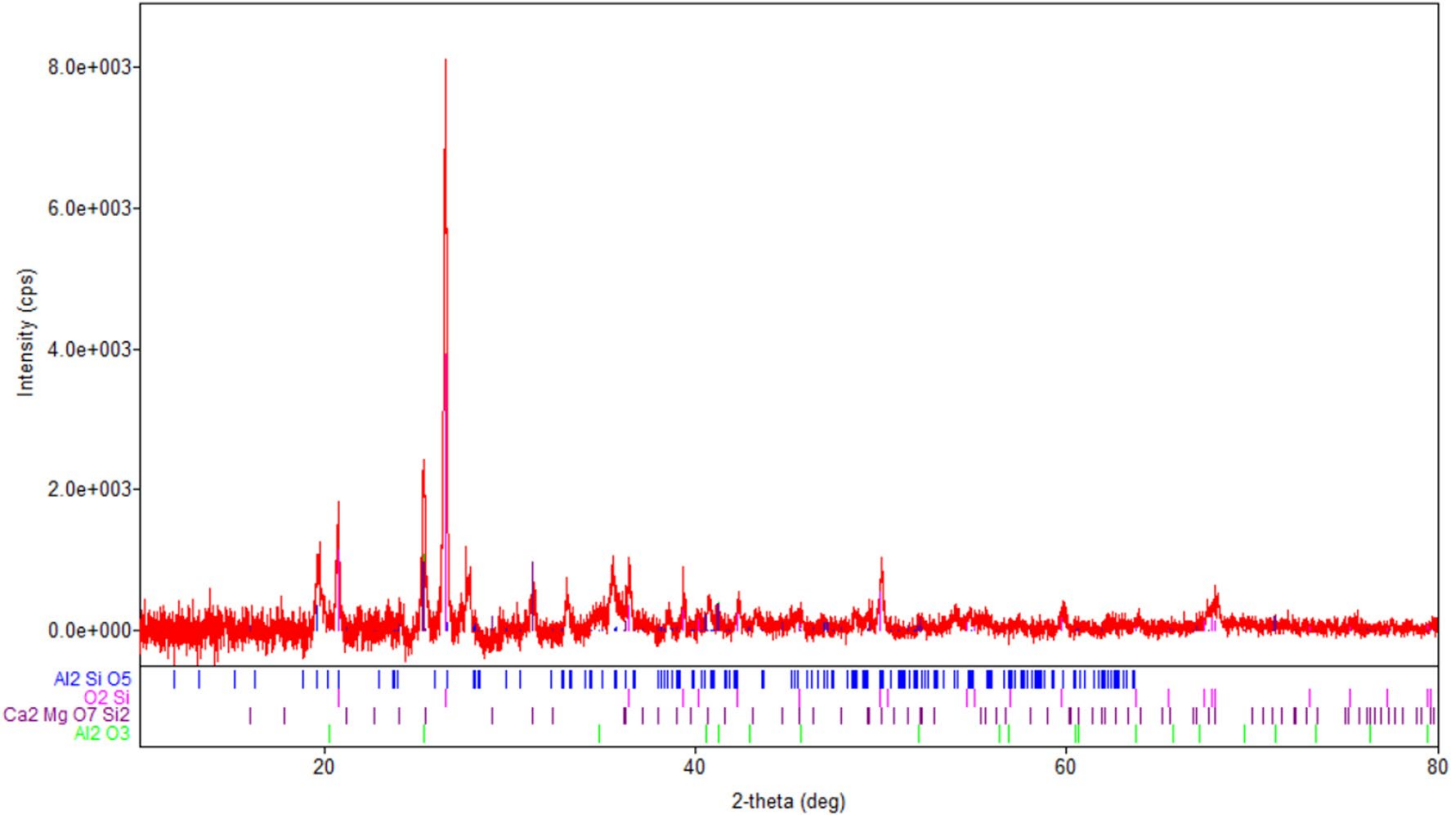
XRD spectra of fly ash sample.

The XRD spectrum (Fig. 4) shows that the fly ash sample has a complex composition. Four crystalline phases have been identified whose characteristics are presented in Table 2.

**Table 2:** The phase compositions of fly ash sample.

Crystalline component	COD card number	Mean crystallite size (nm)	Content (%)
Al_2_SiO_5_ (dialuminum silicate oxide)	1010329	14.6	25
SiO_2_ (quartz)	9009666	27.5	35
Al_2_O_3_ (corundum)	1010951	107.3	37
Ca_2_MgSi_2_O_7_ (akemanite)	9006450	2.8	4

From the data presented in the Table 2 indicates that fly ash sample used in the preparation of the adsorbent is not non-hazardous solid waste^22^.

**Figure 5:**
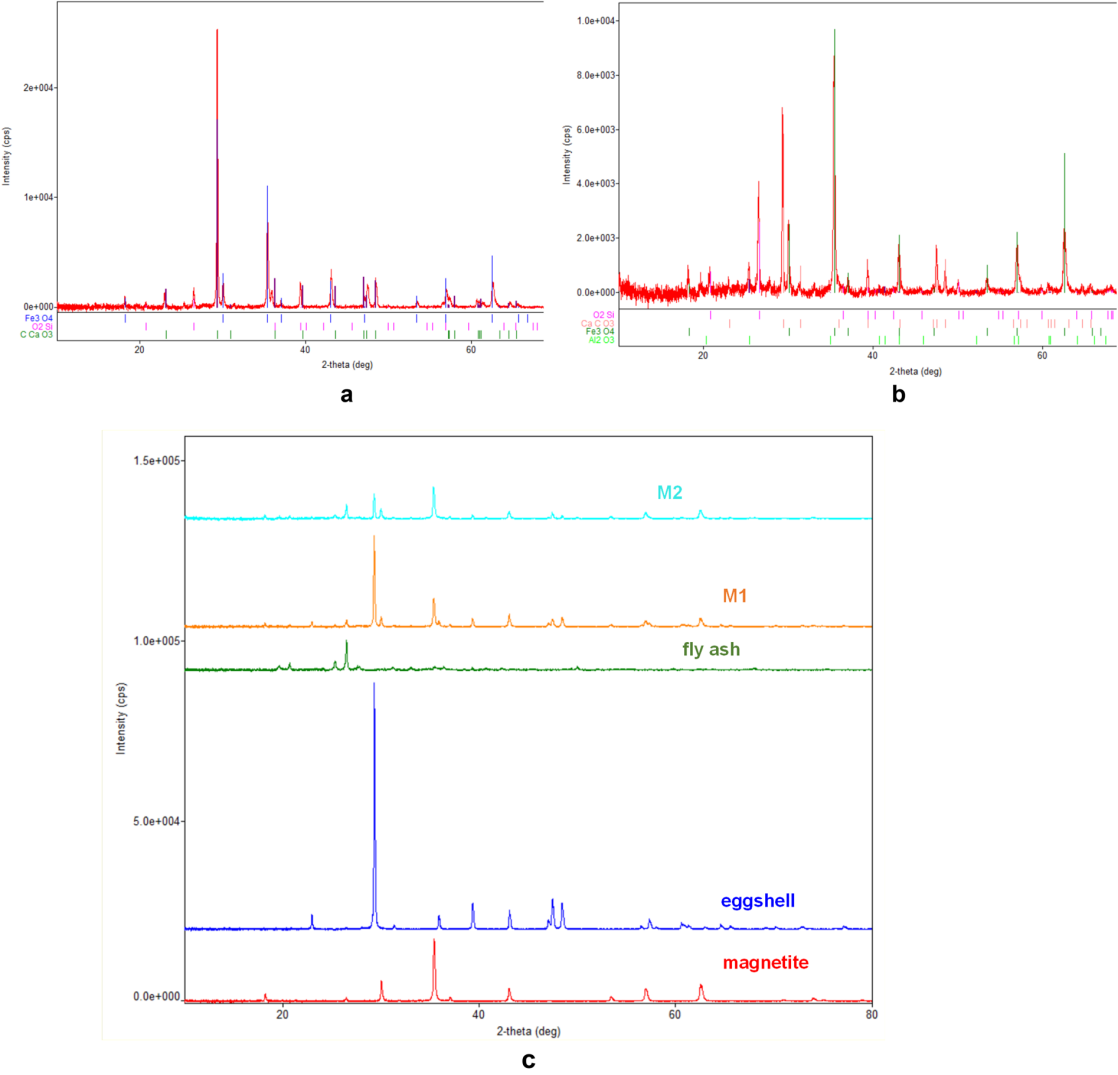
(**a**) XRD spectra of **M1**. (**b**) XRD spectra of **M2**. (**c**) XRD spectra of magnetite, eggshell, fly ash, M1 and M2.

In the XRD spectra of **M1** (Fig. 5a) are visible the diffraction peaks characteristic of the crystalline phases existing in the eggshell (calcite CaCO_3_), the fly ash (but only those of the phase with more intense peaks, quartz SiO_2_), and magnetite (Fe_3_O_4_). Because the eggshell is mixed in a larger proportion than the other two components, the CaCO_3_ peaks are the most intense.

Analyzing the XRD spectrum obtained for **M2** (Fig. 5b), the crystalline phases that can be identified are: magnetite Fe_3_O_4_, calcite CaCO_3_, quartz SiO_2_, and corundum Al_2_O_3_. The most intense are the magnetite peaks. In this sample, because the ash is mixed in a larger proportion, the SiO_2_ peaks are more intense and another crystalline phase present in it (Al_2_O_3_) becomes visible in the spectrum.

Figure 5c shows the overlapping XRD spectra of M1, M2 and raw materials (magnetite, fly ash and eggshell). In both M1 and M2, the phase peaks of individual components can be observed.

However, due to the different materials crystallinity, only the most intense peaks appear by overlap. Also, the phase diffraction lines (Figs. 2, 3, 4, 5) are no longer visible.

#### SEM micrographs

The surface morphology and particle size of raw materials and adsorbent were investigated through SEM technique. The micrographs are presented in Figs. 6, 7, 9, 10, 11, 12, 13 and 14.

**Figure 6:**
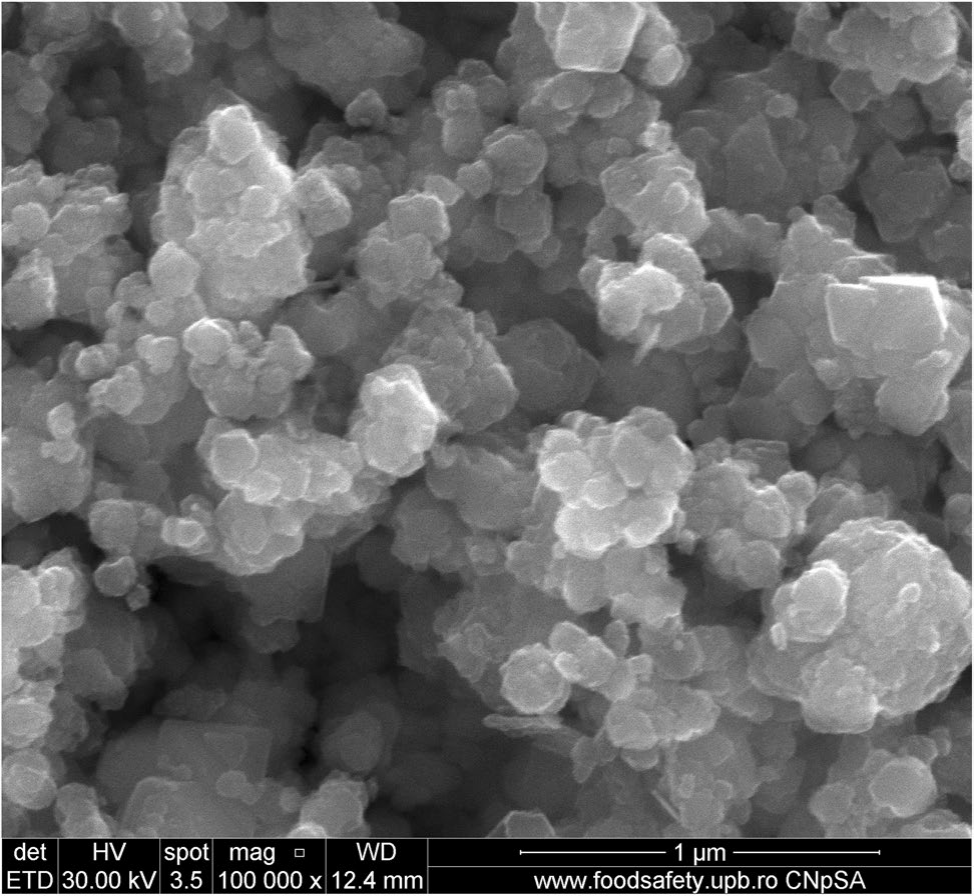
Two-dimensional image of the **magnetite** particle obtained by the SEM technique.

**Figure 7:**
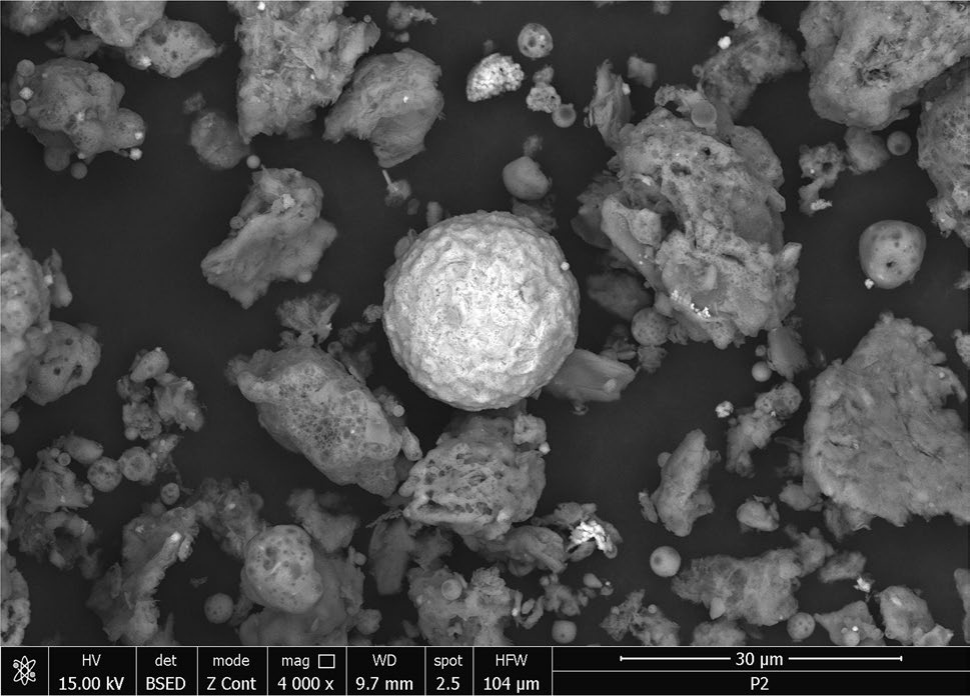
Two-dimensional image of the **ash fly** particle obtained by the SEM technique.

The SEM image of magnetite (Fig. 6) suggest that particles are of nanometric dimensions (with the average size about 21 nm), uniform and with a cubic structure^25,42,43^.

Figure 7 shows that the fly ash particles are of porous spherical shapes with different sizes as well as porous irregularly or angularly shaped particles^16,44^. As can be seen in (Fig. 7) the surface of the ash sphere is irregular and has streaks due to the mechanical and thermal stress.

**Figure 8:**
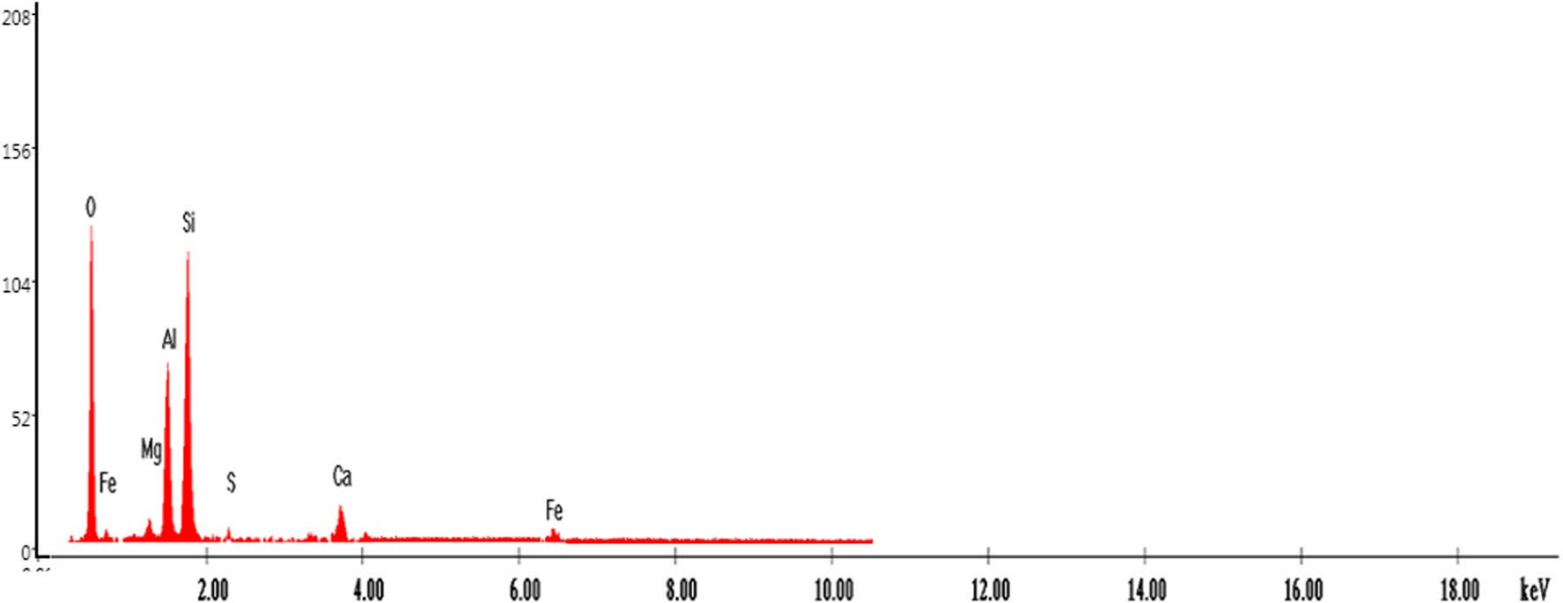
EDS spectra of fly ash sample.

Figure 8 presents the elemental composition of the ash fly determined through EDX analysis.

According to the data from EDX (Fig. 8), there are only seven elements which are predominant in sample: aluminum, iron, magnesium, calcium, silica, oxygen and sulphur^16,45^.

**Figure 9:**
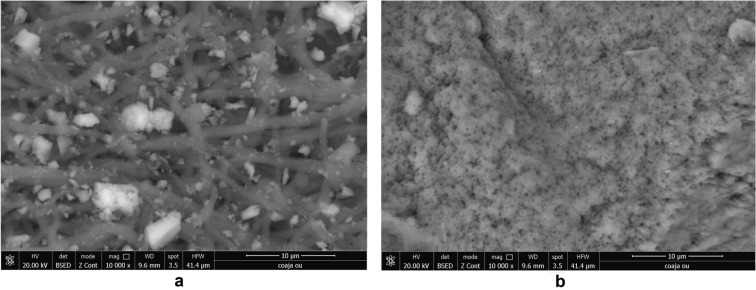
(**a**) Two-dimensional image of the **eggshell** particle obtained by the SEM technique. (**b**) Two-dimensional image of the **eggshell** particle obtained by the SEM technique.

SEM micrograph of eggshell sample (Fig. 9a,b) indicates a different size (about 100 nm) irregular crystal on multihole surface structure^36,40^.

**Figure 10:**
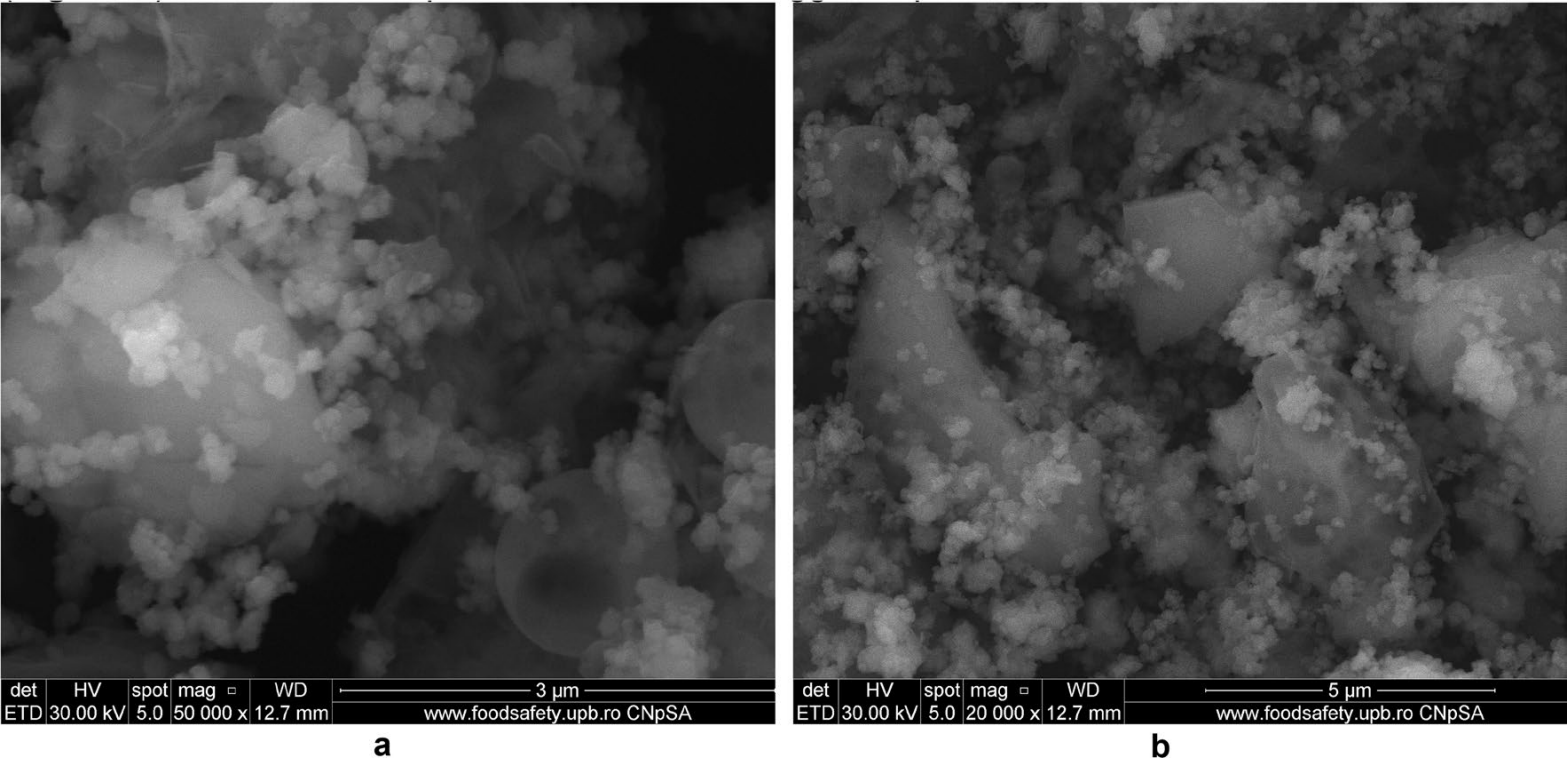
(**a**) Two-dimensional image of **M1** particle obtained by the SEM technique (magnitude 3 µm). (**b**) Two-dimensional image of **M1** particle obtained by the SEM technique (magnitude 5 µm).

The morphology of M1 (Fig. 10a,b) indicates the presence of agglomerations of particles of different sizes in the nano field, spherical shape, cubic shaped and irregular crystal structure sizes, suggesting a good connectivity between them.

Also, the (Fig. 10b) indicates that the cubic-shaped particles characteristic of magnetite (Fig. 6) loaded into the pores of the ash and eggshell particles.

**Figure 11:**
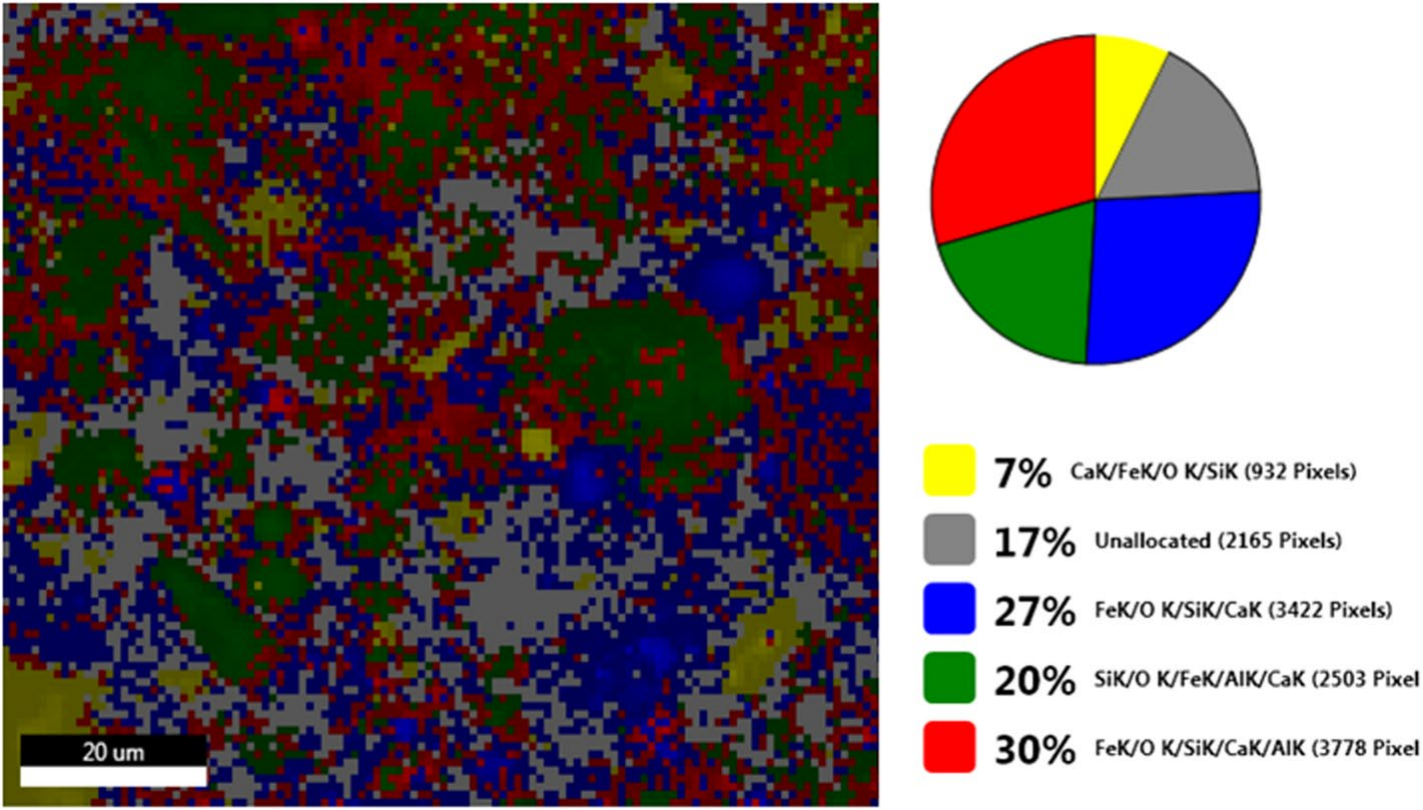
SEM M1- Live map.

The Fig. 11 shows the live map for M1 and the distribution of the identified elements.

**Figure 12:**
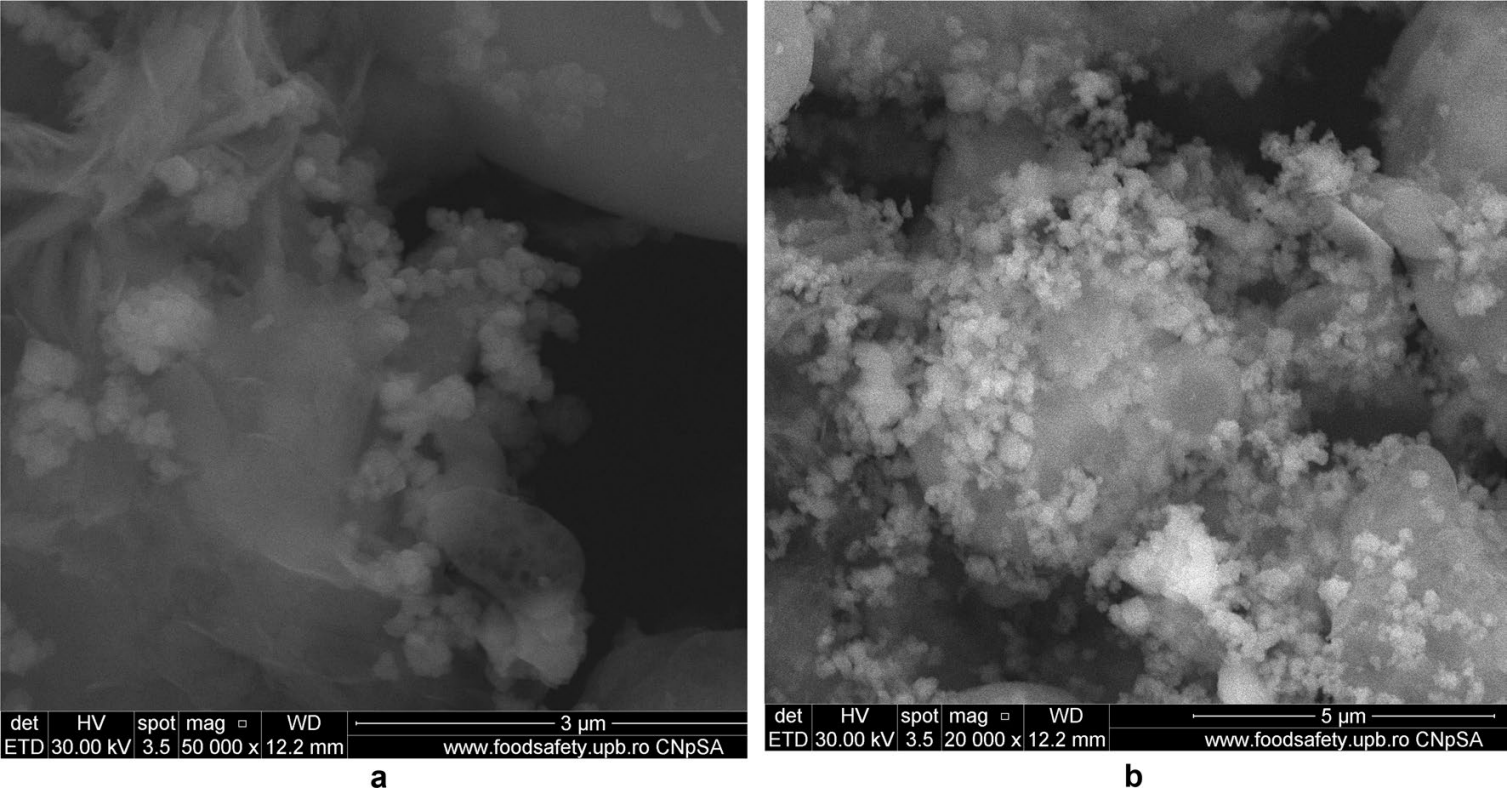
(**a**) Two-dimensional image of **M2** particle obtained by the SEM technique (magnitude 3 µm). (**b**) Two-dimensional image of **M2** particle obtained by the SEM technique (magnitude 5 µm).

The SEM micrograph of **M2** (Fig. 12a,b) the same agglomerations of particles of different nano-sizes, spherical shape, cubic shaped and irregular crystal structure sizes are observed as in the case of SEM graph for **M1**.

**Figure 13:**
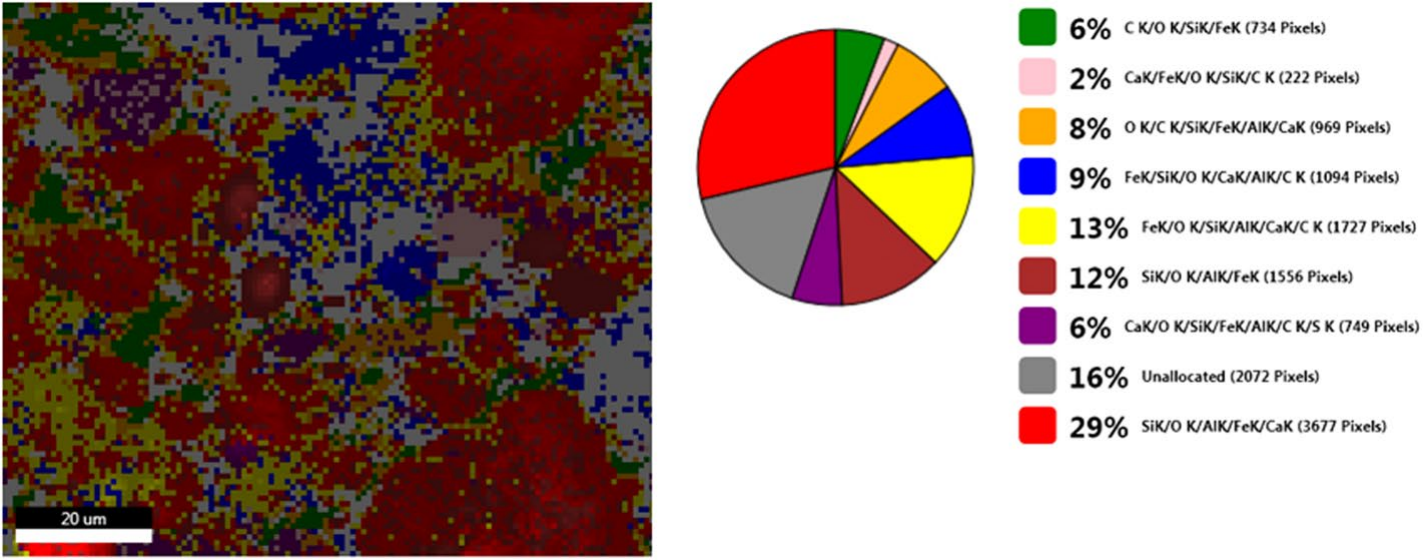
**M2** SEM—Live map.

The Fig. 13 shows the live map for **M2** and the distribution of the identified elements.

The comparative analysis of the Fig. 13 showing Live map for M2 and M1 (Fig. 11) highlights the presence of differences regarding the proportion of identification elements in the two samples, due to the different molar ratio between eggshell and ash.

**Figure 14:**
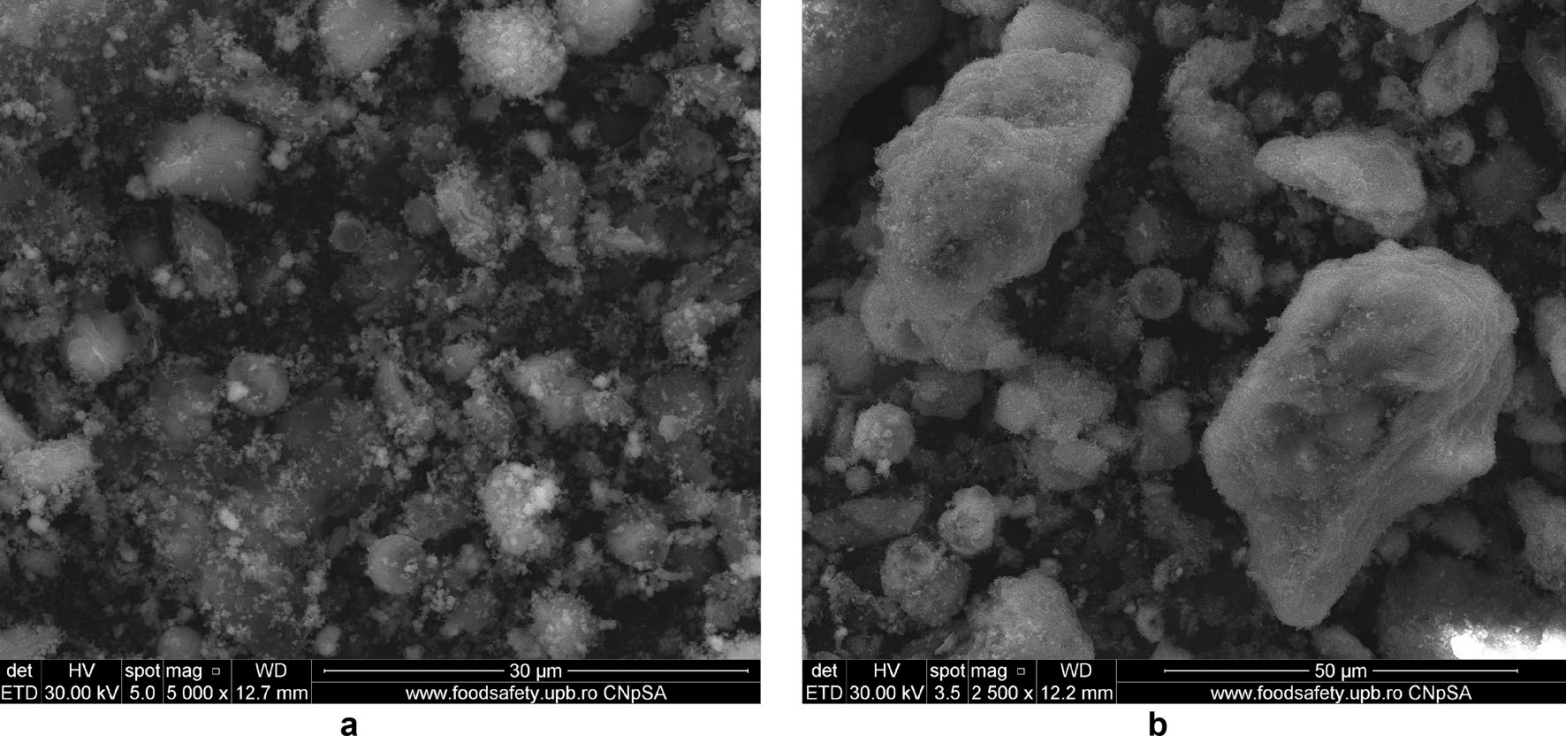
(**a**) Two-dimensional image of **M1** particle obtained by the SEM technique (magnitude 30 µm). (**b**) Two-dimensional image of **M2** particle obtained by the SEM technique (magnitude 50 µm).

In the Fig. 14a can be observed a larger number of spherical particles characteristic of district heating ash, as a result of the change in the ratio between the two wastes (eggshell:ash = 3:1), loaded with magnetite particles. At the same time, in SEM the micrograph for M1 (Fig. 14b) is much more obvious the multihole structure of the eggshell.

The analysis of the SEM micrograph (Fig. 14a) of the M1 sample (in which the eggshell component is predominant) indicates that the multi porous structure of the eggshell is loaded with both the cubic-shaped particles of the magnetite and the spherical ones belonging to the ash sample. This aspect is much more visible in the case of SEM micrograph of the M2 sample (Fig. 14b), considering the fact that in this ash is found in the majority proportion (magnetite:eggshell:ash = 1:1:3).

This result suggests that through the procedure of mechanical alloying in the mill with high energy balls were achieved simultaneously:

1. reducing the particle size of magnetite, ash and eggshell;
2. individual functionalization of each waste (eggshell, ash) with magnetite particles;
3. a new, nanosized material in which the double functionalization of the eggshell with ash particles functionalized with magnetite was achieved simultaneously with the loading of the pores of the eggshell surface with the magnetite particles.

By modifying the structure of the two wastes from the composition of the newly obtained material (decreasing the number of pores) leads to increased surface areas, confirmed by the results of the BET analysis (Table 1) and implicit sorption sites suggesting an improvement of adsorbent properties.

#### FT-IR studies

**Figure 15:**
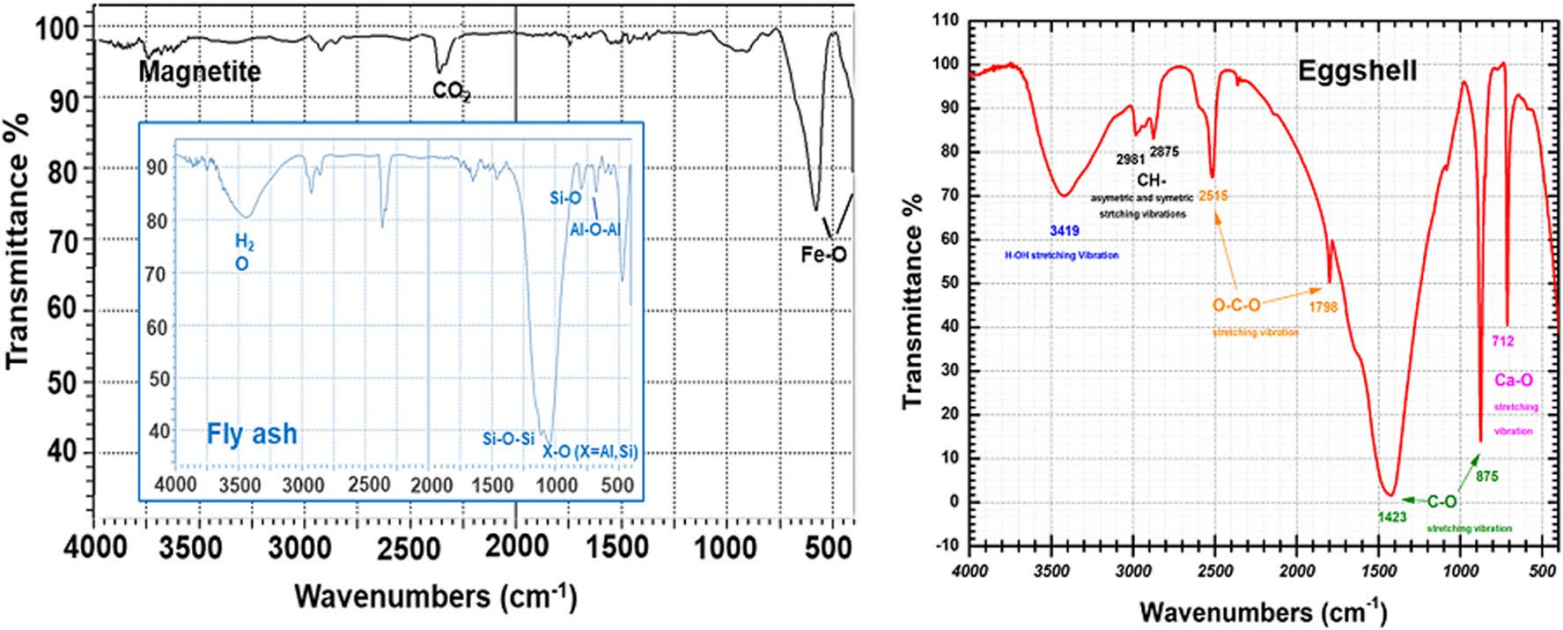
IR spectra for adsorbent raw material samples (magnetite, eggshell and fly ash).

Figure 15 shows the IR spectra for EFM adsorbent raw materials (magnetite, fly ash and eggshell).

**Figure 16:**
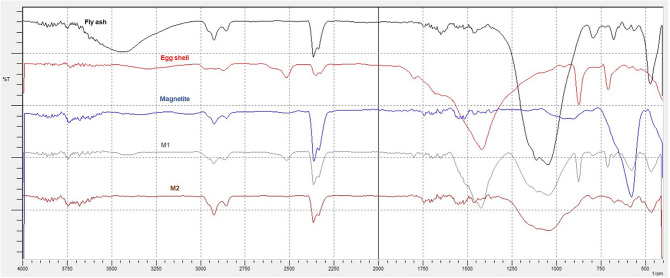
FT-IR spectra of adsorbent **(**both molar ratios**: M1** and **M2**) and its raw materials.

FT-IR spectra for EFM engineered adsorbent are presented in Fig. 16.

The FT-IR spectra for adsorbent (at the both molar ration: M1 and M2) presents the vibrational bands characteristic of magnetite at 589 and at 432 cm^−1^associated with Fe–O stretch vibration^46^. The peaks assigned to the fly ash component: at 588 cm^-1^ Ca O group, at about 670 cm^−1^ attributed to the Al–O–Al bending vibration, at 1100 cm^−1^ is associated with X–O (X = Al, Si) and asymmetric stretching vibrations and band at 830 cm^−1^ specific to AlO_4_ coordination^16,22,47–49^. In addition, in the adsorbent FTIR spectra (Fig. 16) were found the characteristic IR bands eggshell component (Fig. 15). Thus, peak at 712 cm^−1^ (correspond to CaO stretching vibration), peaks at 875 and 1423 are attributed to C–O stretching vibration. The bands at 1798 and 2515 cm^−1^ are associated with O–C–O and peaks at 2875 respectively at 2981 cm^−1^ are due to CH– symmetric and asymmetric stretching vibration^15,31,50^. The position of O–H peak at 3740 cm^−1^ indicates the presence of moisture and water molecules^15,22^. As expected, the intensity of the peaks differs in M1 and M2, due to the different molar ratio between two of the raw materials that are part of the adsorbent component (fly ash and eggshell). These results are in close agreement with the literature and theoretical values confirms the presence of magnetite, fly ash and eggshell in adsorbent (at both molar ration: M1 and M2).

#### Thermogravimetric analysis

**Figure 17:**
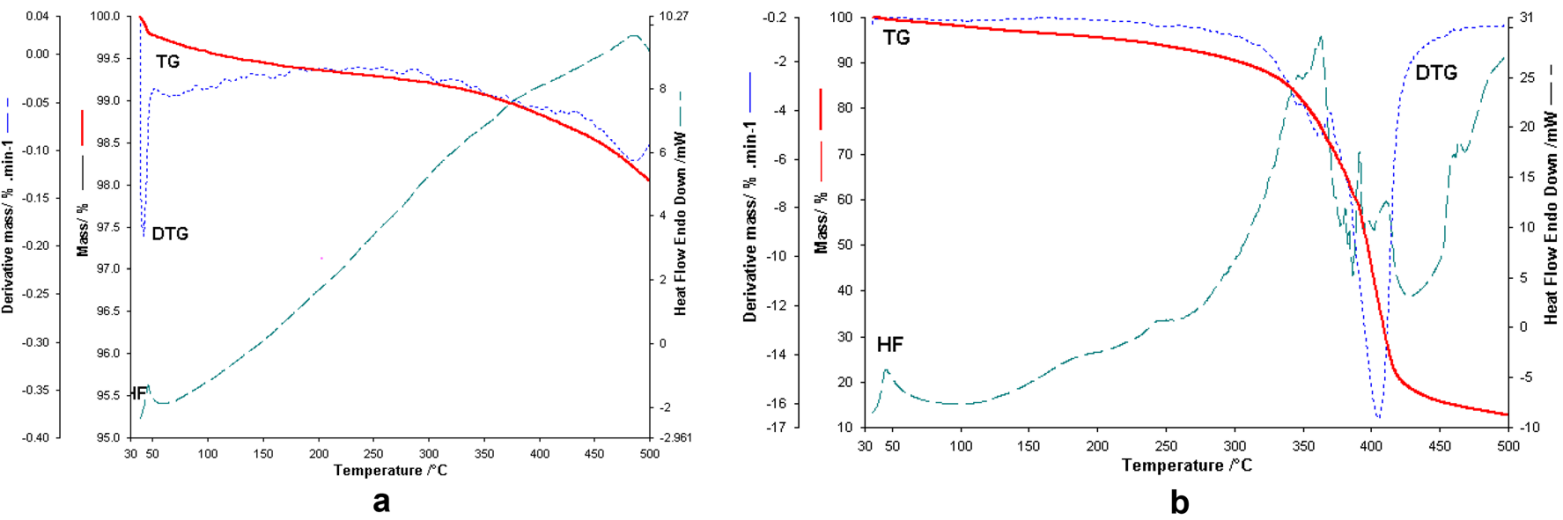
(**a**) Thermogravimetric analysis of the fly ash sample in the range of 30–500 °C with a heating rate of 10 °C/min in open aluminum crucibles in the air atmosphere. (**b**) Thermogravimetric analysis of the eggshell with a heating rate of 10 °C/min up to 500 °C.

Figure 17a presents the thermal analysis results for fly ash sample.

The thermal analysis performed in the interval 30–500 °C highlighted two stages of decomposition. The first stage takes place in the range of 30–49 °C with a loss of 0.22% of the sample mass. This decomposition can be attributed to water loss. This process is visible in the DTG curve with a maximum at 45.5 °C, but also on the Heat Flow curve with a maximum at the same temperature and characterized by an exothermic process with ΔH = − 12.44 J/g. The second process presents a continuous thermal decomposition with a maximum observable on the DTG and HF curve at 480 °C, characterized by an exothermic effect. The decomposition does not end in the studied interval. The total weight loss is 2% of the sample mass^16^.

The thermal analysis, in the range of 30–500 °C, performed for the eggshell sample (Fig. 17b), revealed a complex thermal decomposition. This decomposition has several stages that are difficult to separate. It is known that, in addition to inorganic calcium carbonate compounds, in the eggshell are present a multitude of organic components such as: proteins as main constituents, small amounts of carbohydrates and lipids^51,52^.

At the same time, uronic acid is also present, which plays an important role in the resistance of the shell, such as sialic acid in very low concentration and two glycosaminoglycans, including hyaluronic acid, as well as a copolymer consisting of chondroitin sulfate-dermatan sulfate. There is also limited information on variations in nitrogen concentrations and the amino acid composition of the eggshell. A better understanding of the chemicals present in the composition of the eggshell is very important for its application in various fields, including for the purpose of absorbent material.

The analysis of the TG curve highlights three hardly separable decomposition stages, the last of which is characterized by a complex multistage decomposition process. It is observed that in the interval 30–100 °C which can be attributed to dehydration, followed by the loss of crystallization water in the range 100–266 °C (4.8% of the sample mass) and then the complex decomposition of organic components in different stages depending on their stability until at 500 °C^15^.

The last decomposition stage results in the loss of 80% of the total mass of the sample. Over 500 °C the decomposition of the inorganic component takes place, namely Ca carbonate. It can be seen that in the analyzed sample the weight of inorganic component is relatively small, namely 12.2% of the sample mass. During the decomposition stage from the interval 266–500 °C several maxima are observed on the DTG curve, which led us to conclude that simultaneous decompositions of several organic compounds take place, observing maxima at 345, 363, 374, 403, 408, 412, 430, 466 and 470 °C. The same main decomposition steps are faintly visible and the HF curve with processes in most cases exothermic. At temperatures higher than 266 °C and on this curve are visible several processes, most of which are exothermic, which can be attributed to the oxidation of organic compounds and their decomposition. The residue left after the thermogravimetric study (performed up to 500 °C) is calcium carbonate^15,53^.

Subsequently, the mixture of the two wastes was analyzed in the two molar ratios:eggshell:fly ash = 3:1 and eggshell:fly ash = 1:3, respectively.

**Figure 18:**
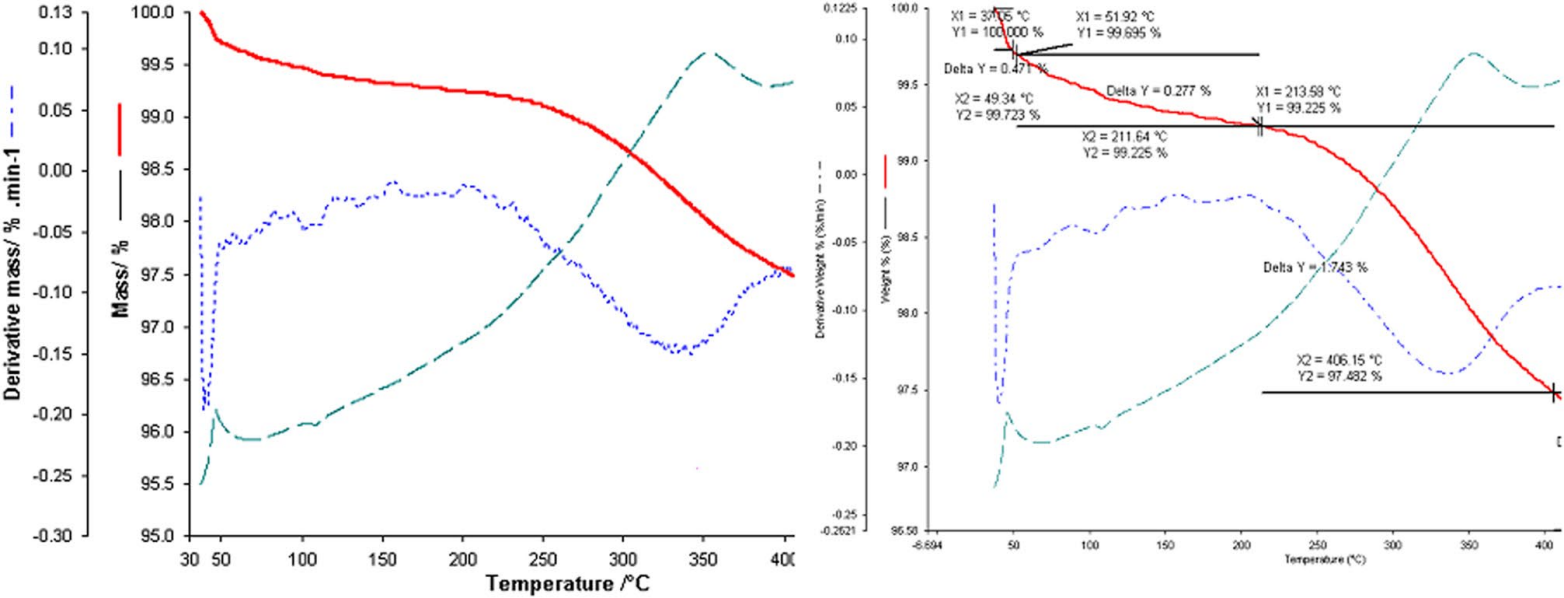
Thermogravimetric analysis for eggshell: fly ash binary mixture in a 1: 3 mass ratios obtained in the range of 30–500 °C.

The profile of thermogravimetric analysis for the binary mixture eggshell:ash fly in a 1:3 mass ratio performed in the range of 30–500 °C is depicted in Fig. 18.

The thermal analysis performed in the case of the binary mixture of eggshell and ash, in a 1:3 molar ratios, highlights the decomposition stages of the two components. Namely, the stage of water loss within the ash is visible, to which is added the loss of moisture observed in the case of the eggshell. On the HF flow is visible the exothermic process with a maximum of 45 °C and a ΔH = − 17.023 J/g which represents a sum of the two processes mentioned above. Two other processes are visible on the TG curve, one in the temperature range, 51–213 °C, with a mass loss of 0.27% of the sample mass. Then followed by a loss of 1.74% in the temperature range 213–405 °C. The thermal decomposition continues even above this temperature and the decomposition process was not completed in the studied temperature range.

**Figure 19:**
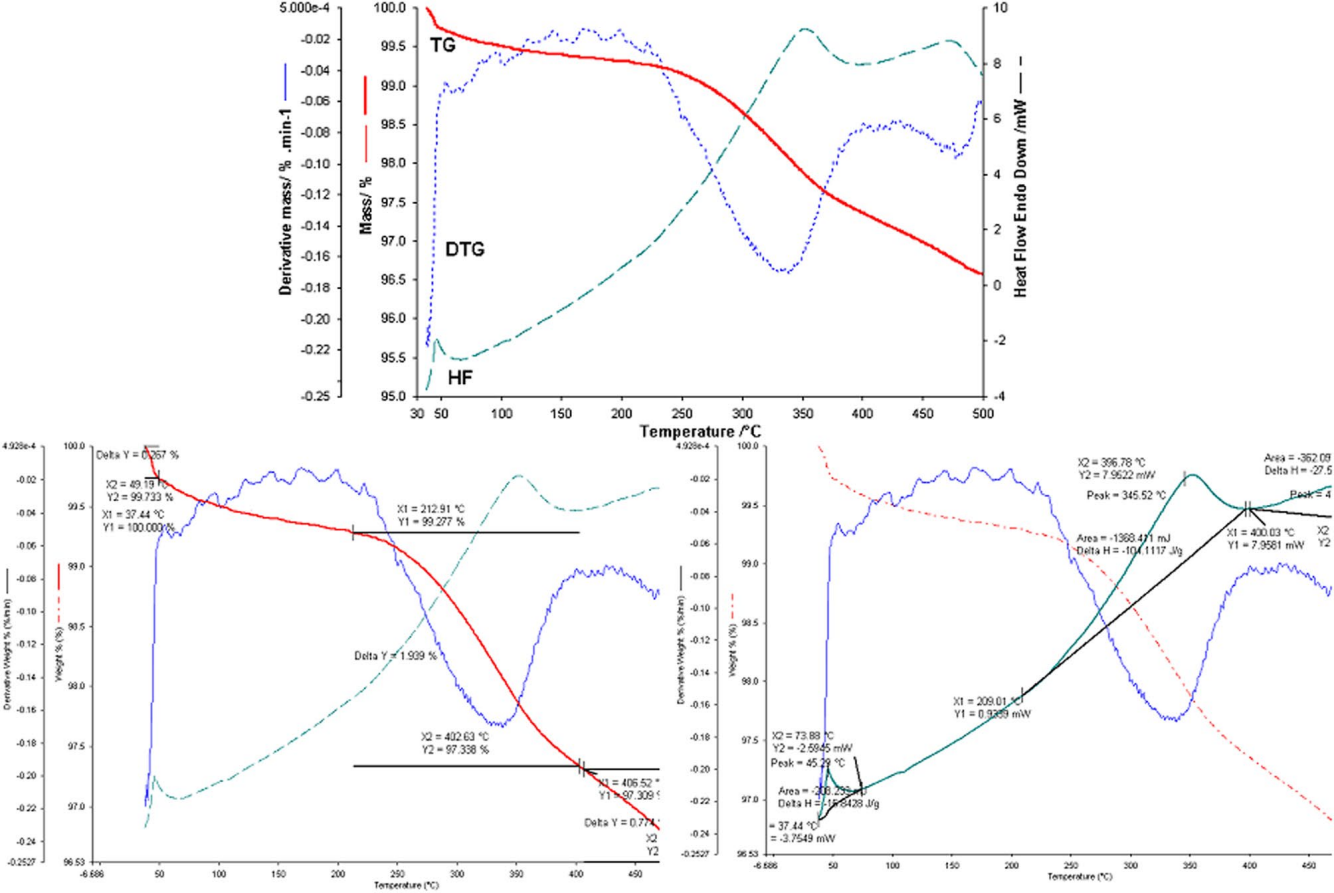
Thermogravimetric analysis for eggshell: fly ash binary mixture in a 3:1 mass ratio obtained in the range of 30–500 °C.

A thermogravimetric study was performed for the same binary mixture but in the eggshell:fly ash = 3:1 molar ratio. The results are presented in the next figure (Fig. 19).

In the case of the thermal analysis of the binary mixture of eggshell and ash in a 3:1 molar ratio, the decomposition stages and the thermal behaviour of the individual components in correlation with the mixing ratio are very clearly visible.

#### Magnetic measurements

**Figure 20:**
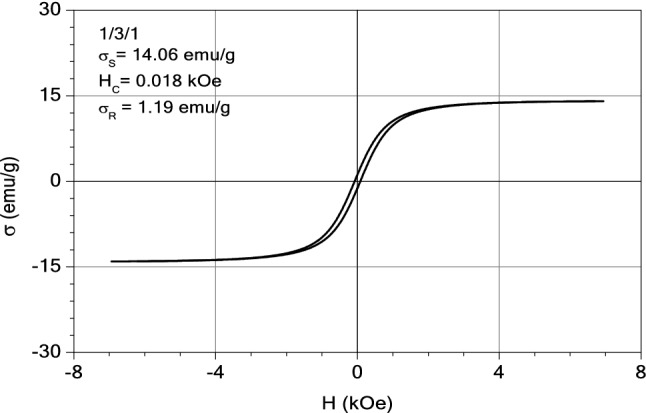
The hysteresis loop of sample M2.

**Figure 21:**
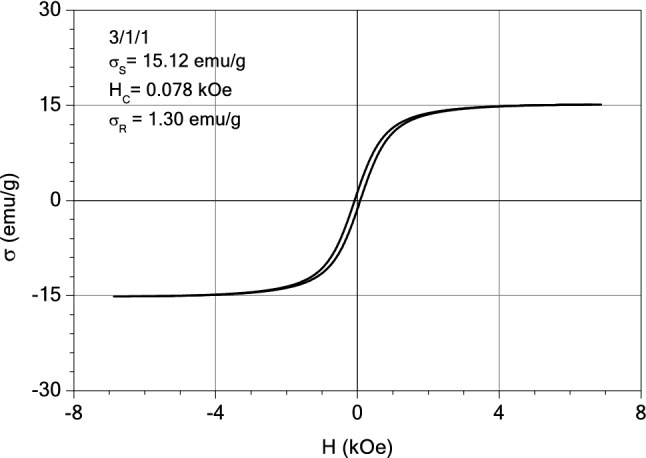
The hysteresis loop of sample M1.

**Figure 22:**
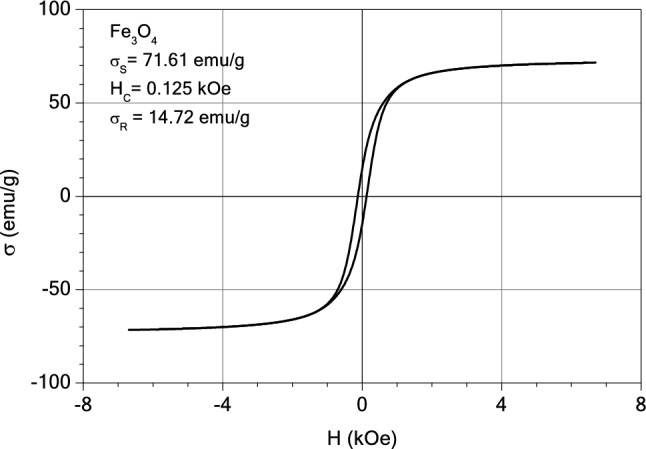
The hysteresis loop of magnetite.

The magnetic properties of the samples: magnetite, M1 and M2 were investigated with an induction hysteresis-graph at low frequency driving field (50 Hz)^54^. And the hysteresis loops are presented in Figs. 20, 21 and 22. It was found that the samples reveal ferromagnetic behaviour and from the measured hysteresis loops the saturation magnetization ($\sigma_{S}$), the coercive field (*H*_*c*_) and the remnant magnetization ($\sigma_{R}$) were determined. The results are presented in Table 3.

**Table 3:** The values of coercive field (*H*_*c*_) and remnant magnetization ($\sigma_{R}$) of M1, M2 and magnetite sample.

Sample	$\sigma_{S}$ (emu/g)	H_C_ (kOe)	$\sigma_{R}$ (emu/g)	$\sigma_{R} /\sigma_{S}$
M2	14.06	0.081	1.19	0.084
M1	15.12	0.078	1.30	0.086
Magnetite	71.61	0.125	14.72	0.205

As expected, the largest value of the saturation magnetization is that of the sample consisting entirely of magnetite. By diminishing the content of ashes from the thermal power station (from three parts in M2 sample to one part in sample M1) a small increase of the saturation magnetization was observed, from 14.06 to 15.12 emu/g (see Table 3). This can be explained by the presence of diamagnetic compounds within the ashes of the thermal station, the decrease of which led to the increase in the saturation magnetization of the sample M1, as compared to the sample M2. All three samples have small values of the remnant ratio, $\sigma_{R} /\sigma_{S}$, which is an indication of the ease with which the magnetization reorients to the nearest easy axis magnetization direction after the remove of magnetic field.

**Figure 23:**
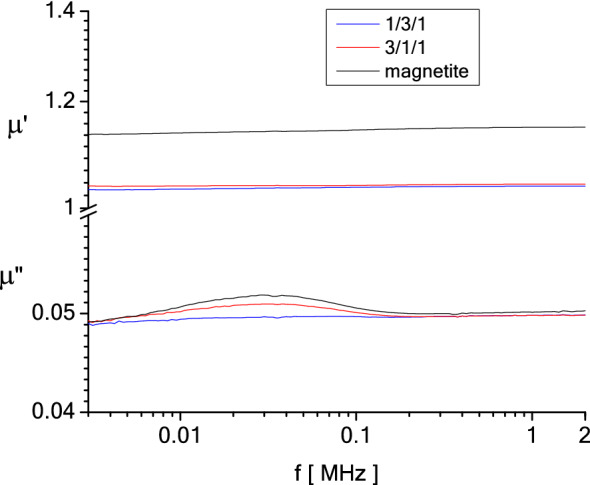
Frequency dependence of the magnetite, M1, M2 of the complex magnetic permeability.

The dependencies on frequency of the complex magnetic permeability of the samples, $\mu \left( f \right) = \mu^{\prime}\left( f \right) - i\mu^{\prime\prime}\left( f \right)$, measured at room temperature, over the frequency range 3 kHz to 2 MHz are presented in Fig. 23. The measurements were performed using an Agilent LCR-meter (E-4980A type) in conjunction with a coil containing a vial in which the samples were placed. Details on the method of measurements of the real, $\mu^{\prime}\left( f \right)$ and imaginary, $\mu^{\prime\prime}\left( f \right)$ components of the complex magnetic permeability are given in a previous study^55^.

In the frequency range in which the measurements were made, samples M1 and magnetite exhibits visible relaxation peaks of $\mu^{\prime\prime}\left( f \right)$, at the frequency of 30 kHz. Even if the M1 sample and the M2 sample have the same amount of magnetite, due to the diamagnetic compounds in the fly ash, the relaxation peak of the M1 sample is very attenuated (little visible, almost missing).

Given the small size of the magnetite particles in the samples (on the order of tens of nanometers), they do not have a multi-domain magnetic structure. Thus, the only magnetic relaxation process, measurable in the radio frequency field, is the Neel relaxation process. The Néel relaxation time, $\tau_{N}$ is given by Eq. (15)^56^

15 $$\tau_{N} = \tau_{0} \exp \left( {\frac{Kv}{{k_{B} T}}} \right)$$

where *K* is the effective anisotropy constant of the material from which the magnetic particles are made of, *v* is the magnetic volume of particles, *k*_*B*_ is the Boltzmann’s constant, *T* is the temperature and $\tau_{0}$ is a constant in order of *10*^*–9*^* s*^56^*.*

Assuming that the frequency dependence of the complex magnetic permeability,$\mu \left( f \right) = \mu^{\prime}\left( f \right) - i\mu^{\prime\prime}\left( f \right)$ obeys the Debye dispersion relations, then the frequency corresponding to the maximum of $\mu^{\prime\prime}\left( f \right)$ is correlated with the relaxation time by the relation, $2\pi {\kern 1pt} f\tau = 1$. For measurements at room temperature, with *f* = *30 kHz*, the magneto-crystalline anisotropy constant of magnetite, *K* = *1.1* × *10*^*4*^ J m^−3^ and $\tau = \tau_{N}$, under assumption of spherical shape of particles, one gets a magnetic diameter of the magnetite particles, *d* = *18.2* nm. This value compares favourably with the values measured by SEM and X-ray diffraction.

#### Adsorption properties

##### Effect of adsorbent dosage

**Figure 24:**
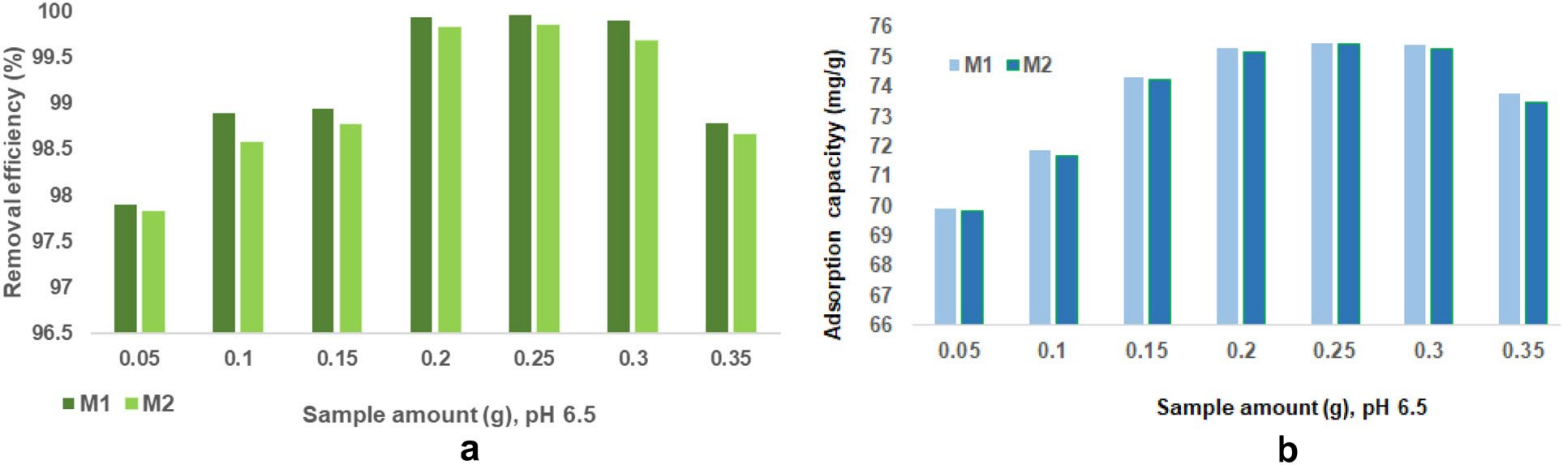
(**a**) The relationship between different material dosage and the cadmium removal efficiency. (**b**) The relationship between different material dosage and the cadmium adsorption capacity.

Figure 24a and b show the relationships between different material dosage and the cadmium removal efficiency and respectively adsorption capacity.

According to the Fig. 24a and b, cadmium removal efficiency and adsorption capacity depending on the amount of adsorbent used shows an upward trend (for quantities between 0.05 and 0.25 g), reaches a maximum of 0.25 g adsorbent (99.9% and 75.48 mg/g for M1 and respectively 99.8% and 75.46 mg/g for M2), after which both removal efficiency and heavy metal adsorption capacity gradually decrease with the increase in the adsorbent dose (0.3 g). These results suggest that the increased amount of adsorbent provides a supplement to the free active sites, but after reaching equilibrium, it leads to the formation of agglomerations and consequently to a decrease in the number of available active sites^22,23^.

##### Effect of initial concentration on cadmium removal efficiency

**Figure 25:**
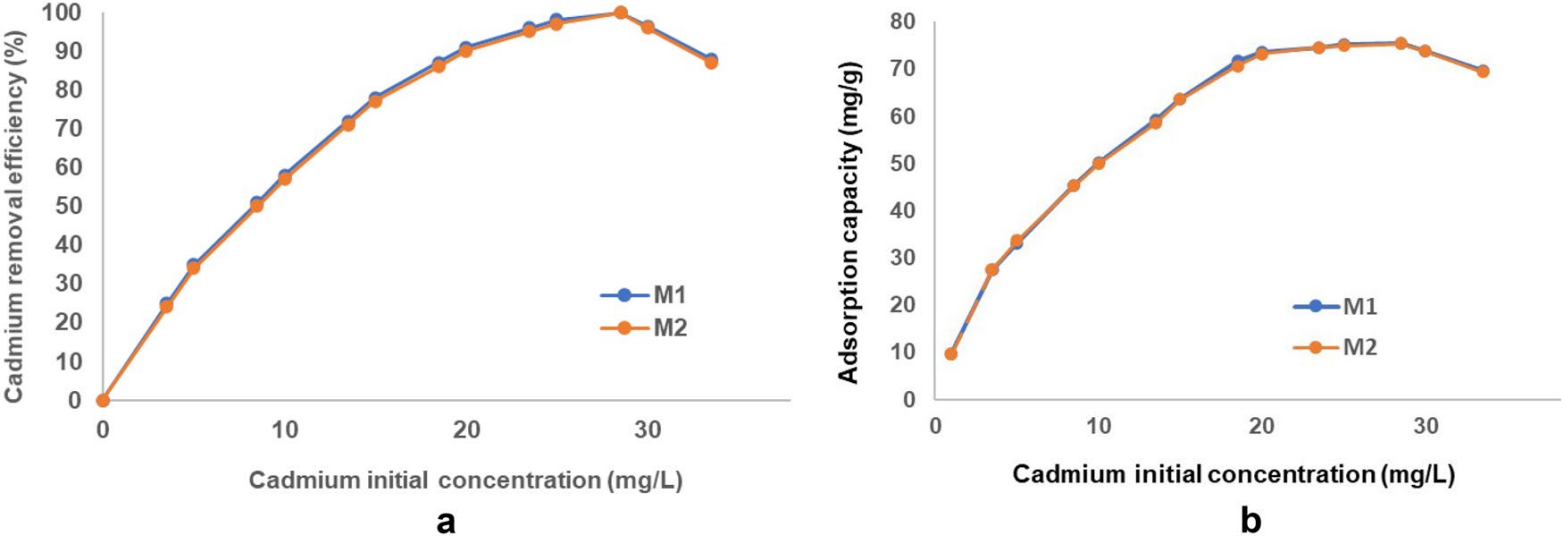
(**a**) Relationship between initial concentration and removal efficiency (%). (**b**) Relationship between initial concentration and adsorption capacity (mg/g).

Figure 25a shows the influence of heavy metal initial concentration on cadmium removal efficiency. It can be seen that removal efficiency shows an upward trend simultaneously with the increase of the initial cadmium concentration in the range 0–33.5 mg/L. The maximum removal efficiency (99,9% for M1 and respectively 99.8% for M2) was reached at a concentration of 28.5 mg/L, after which the decrease in cadmium removal efficiency begins.

According to the Fig. 25b, in the same cadmium concentration range (0–33.5 mg/L), the adsorption capacity shows a similar trend, reaching a maximum at 28.5 mg/L (75.48 mg/g for M1 and respectively 74.46 mg/g for M2), and after which gradually decreases.

These results indicate the initially an increase in the concentration of heavy metal causes an increase in the amount of Cd^2 +^ ions and implicitly in the possibility of interaction with the active sites of the EFM adsorbent. And after reaching equilibrium, the amount of available metal ions is disproportionate compared to the decreasing number of free sites in the adsorbent, causing a decrease in the adsorption efficiency of the new engineered magnetic adsorbent used in the study^15,57^.

##### Effect of pH

The wastewater pH is one of the top parameters with highly influence on the adsorption process efficiency having impact direct on the adsorption rate and adsorption capacity as fluctuations in the pH value of the solute induce changes in the degree of ionization of the adsorptive species and the of adsorbent surface^23,57^.

In this study was investigated the pH influence toward the cadmium removal using the prepared material in the pH range of 3.0–7.0, to avoid the precipitation of Cd(OH)_2_ at pH values > 7^15^.

**Figure 26:**
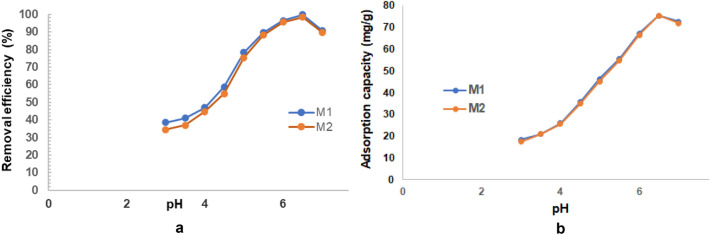
(**a**) Effect of pH variation on cadmium removal efficiency. (**b**) Effect of pH variation on adsorption capacity.

According to the experimental results presented in Fig. 26a and b, the increase in pH value (between pH 3 and pH 6) leads to a significant increase in adsorption efficiency and adsorption capacity. The adsorption efficiency and adsorption capacity reach a maximum value (99.9% and 75.48 mg/g for M1 and respectively 99.8% and 75.46 mg/g for M2) at pH 6.5, after which it decreases slightly. This could be explained as follows: at low pH values is a competition between protons and Cd^2+^ to occupy the active sites of the adsorbent, even if they are available in large numbers. An increase in pH simultaneously leads to a decrease in the competition of protons and electrostatic repulsion forces, which induces an increase in cadmium removal efficiency. At pH > 6.5 the removal efficiency begins to decrease as increased hydroxyl ion generation occurs to the detriment of Cd^2+^ ions. Therefore, the optimal pH 6.5 was chosen for subsequent experiments^15,16,25,58,59^.

##### Effect of contact time

**Figure 27:**
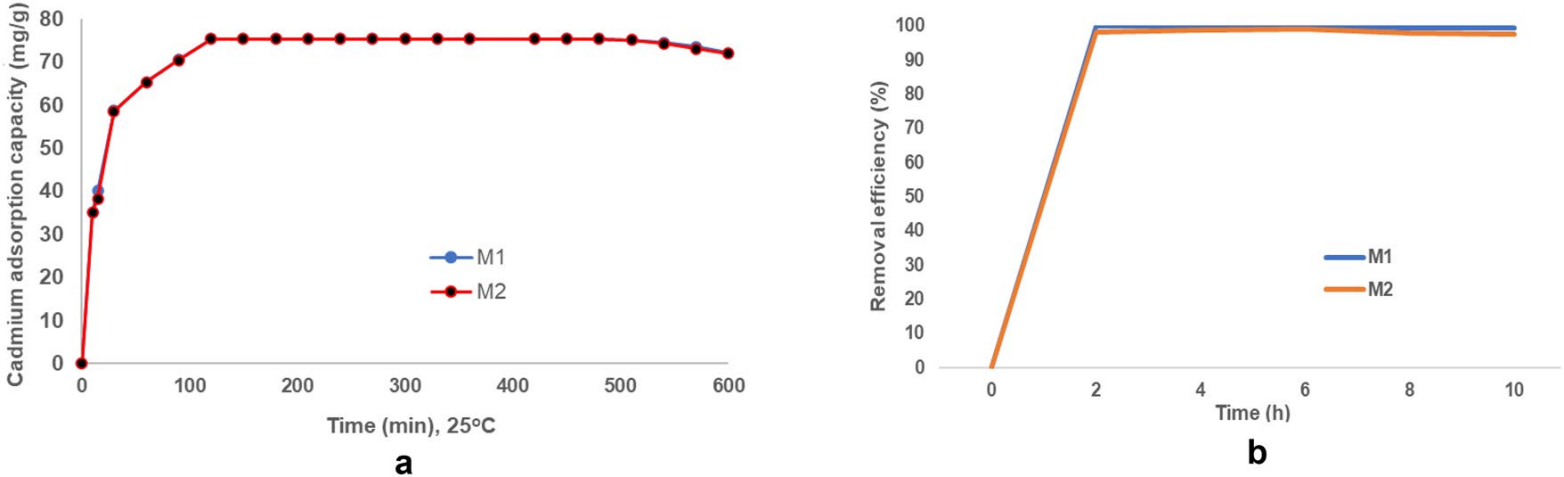
(**a**) Effect pf contact time on cadmium adsorption capacity (mg/g). (**b**) Effect of contact time on cadmium removal efficiency (%).

Figure 27a showed the relationship diagram between the contact time and cadmium adsorption capacity.

It can be observed from the Fig. 27a and b that the increase of the contact time determines an increase of the adsorption capacity and of the removal efficiency respectively. Both reached the maxima at 120 min The maximum of cadmium adsorption capacity was 75.48 mg/g for M1 respectively 75.46 mg/g for M2, and the maximum of removal efficiency was 99.9% for M1 and respectively 99.8% for M2^32^.

This performance can be attributed to the higher surface, the microporous structure that results from the experimental conditions of this study^15^.

The analysis of this diagram indicates that the adsorption of cadmium takes place in three distinct phases:

1. 0–90 min, characterized by adsorption is fast due to the large number of active sites available on the surface of the adsorbent.
2. the second phase, 90–120 min, the adsorption is slower which can be attributed to the diminution of the free adsorbent active sites;
3. phase three:120–330 min, corresponds to the time interval in which there are no more free sites on the surface of the adsorbent and the adsorption has reached equilibrium.

According to the experimental results**,** optimum time in which the adsorption reaches an equilibrium is 120 min and was selected for the next investigations^16,60^.

##### Effect of temperature on absorption process

**Figure 28:**
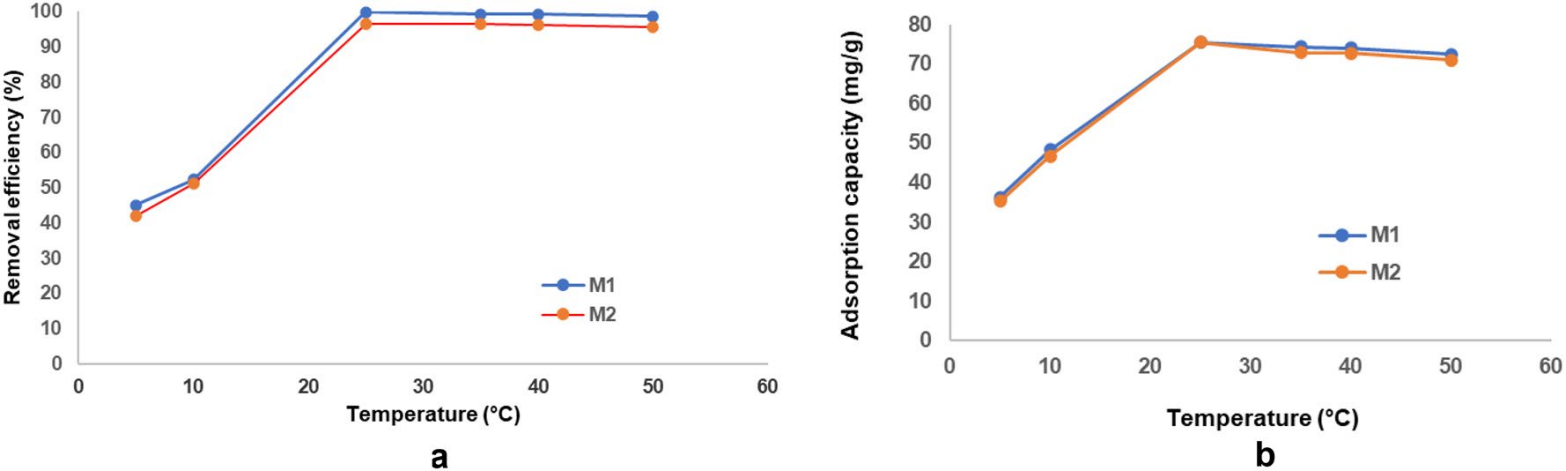
(**a**) Relationship between temperature and heavy metal removal efficiency. (**b**) Relationship between temperature and heavy metal adsorption capacity.

Temperature represents a key parameter in adsorption process. Therefore, the influence of temperature on cadmium adsorption on prepared material in the two different molar ratios (both M1 and M2) was investigated in the range of 5–50 °C (278.15–323.15 K). The cadmium removal efficiency and adsorption capacity increase first and then a very slight decrease occurs with the increase of temperature (Fig. 28a,b).

At 25 °C the maximum removal efficiency is reached (99.89% for M1 and respectively 99.64% for M2). At the same temperature the heavy metal adsorption capacity is maximum of 75.48 mg/g for M1 and 75.43 mg/g for M2. This can be explained by the fact that within this temperature range indicated the favorability for the heavy metal mobilization and thus contact between cadmium and active sites from adsorbent. The relationship between temperature and cadmium adsorption effect indicates that in the range of 5–25 °C the cadmium absorption on prepared material is an endothermic process (physical adsorption). At 25–50 °C the adsorption process becomes exothermic and chemisorption occurs. However, the removal efficiency remains very high even at a temperature of 50 °C (98.78% for M1 and respectively 97.74% for M2).

#### Comparison of cadmium removal efficiency for with other adsorbents

A comparison of cadmium removal efficiency of the newly engineered adsorbent (EFM) with other adsorbents reported in literature is presented in the next table (Table 4).

**Table 4:** Comparison of the removal efficiency of newly nanosized magnetic adsorbent (at both molar ratios: M1 and M2) with the one reported in the literature (selected study) for some adsorbent materials that use the similar waste.

Adsorbent type	Removal efficiency (%)	References
Eggshell	96	^15^
Eggshell	25.56	^36^
Eggshell	93	^33^
Calcined eggshell	99	^40^
Coal fly ash	71	^22^
Magnetite nanoparticles	72	^25^
FeNPs	41.7	^32^
F-ES (eggshell loaded with FeOOH)	94.26	^36^
Polyelectrolyte-coated fly ash (PEFA)	96	^16^
EFM	99.9 (M1) and 99.8 (M2)	This study

The performance of the nanosized adsorbent EFM (at both molar ratios) can be attributed to the higher surface area, the microporous structure that results from the experimental conditions of this study^15^.

#### Comparison of cadmium removal efficiency with the raw materials

**Figure 29:**
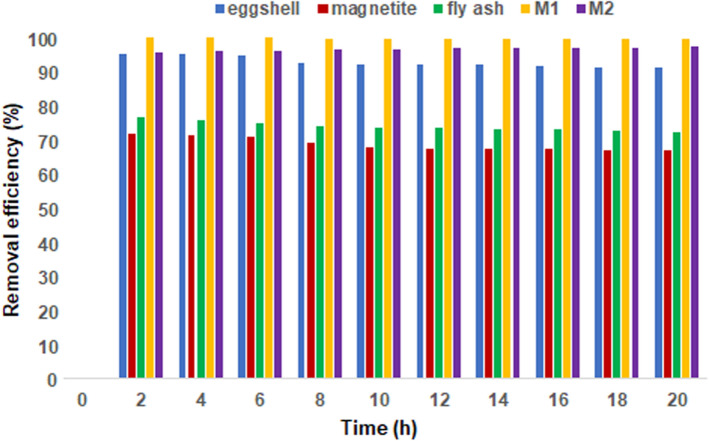
Relationship between adsorbents removal efficiency and contact time.

The removal efficiency of EFM adsorbent compared to that of its raw materials (fly ash, eggshell and magnetite) was investigated as the effect of contact time on the adsorption process. The relationship between the removal efficiency and contact time is presented in Fig. 29. It can be observed that there is an increase in the efficiency of removing heavy metal for all five investigated adsorbents (eggshell, ash, magnetite, M1 and M2) with a maximum of two hours of contact. According to the experimental results presented in Fig. 29, the best cadmium removal efficiency was obtained for M1 (99.89%) and 99.80% for M2, followed by eggshell (95.23%), fly ash (76.31%) and magnetite (71.44%).

Then, a very slight decrease occurs with the increase in contact time. These results confirm the cadmium removal efficiency dependence on the specific surface area and pores (number of available active sites) of the adsorbent used (Table 1).

The maximum cadmium removal efficiencies determined experimentally in this study for the raw materials (eggshell, ash and magnetite) corroborated with the data reported in the literature^15,25,33,61^.

#### Adsorption Isotherms

The absorption mechanism evaluation can be performed through an isotherm adsorbent study. The equilibrium isotherm plays a key role in the investigation of the adsorption behaviour.

Due to their simplicity and convenient accuracy, Langmuir and Freundlich's models are the most commonly used to adjust an adsorption process.

Langmuir models provides information on the interaction between the solute and the monolayer surface of the adsorbent. The main working hypotheses of this model are: (1) adsorbent surface consists of uniform, identical sites distributed on the surface of adsorbent (2) adsorbent process takes place only on the surface of the adsorbent and (3) no contact between adsorbed molecules on the surface of the adsorbent.

Freundlich model is appropriate to monolayer and multilayer adsorption processes on multiphase surfaces. This isotherm gives an expression on adsorbent surface heterogeneity and the variation in the heat of adsorption process. The applicability of the Freundlich model is limited by adsorption processes that take place at high pressures, but this restriction does not apply to the Langmuir model.

These two adsorption isotherm models were applied in order to identify and implement an optimal model that adequately reproduces the experimental results obtained in this study were employed to study the mechanism of cadmium adsorption on the prepared material^60,62^.

The parameters calculated as well the coefficient of correlation (R^2^) for both Langmuir and Freundlich models are presented in the Table 5.

**Table 5:** Parameters of adsorption Langmuir and Freundlich isotherms for cadmium adsorption.

Adsorbent material	Langmuir model					Freundlich model		
	Q_e_	Q_m_	K_L_	R_L_	R^2^	K_F_	*n*	R^2^
M1	75.45	88.29	0.0221	0.33	0.9887	3.4164	1.2481	0.9983
M2	75.43	88.27	0.0219	0.34	0.9885	3.4141	1.2478	0.9984

As shown in the Table 5 both models fitted well for the experimental results. The maximum capacities calculated are close to the values for each component of the prepared adsorbent material (magnetite, eggshell and fly ash) and maximum capacities obtained at equilibrium (Table 5)^22,23,57,63,64^. However, according to the values of the correlation coefficient, R^2^, the behaviour of cadmium absorption suits better with Freundlich model (the higher correlation coefficient) suggests that the adsorption for cadmium ions was a multi-molecular layer adsorption process. The values for the Langmuir constant, K_L_, or equilibrium parameter for absorbent (the both molar ratios, M1 respectively M2) falls within the range 0 < R_L_ > 1 indicated a favourable adsorption process. Moreover, Freundlich dimensionless constant n values having greater than 1 suggests a favourable adsorption process that occurs on the investigated EFM adsorbent heterogeneous surfaces^62,65,66^.

#### Adsorption kinetic study

**Figure 30:**
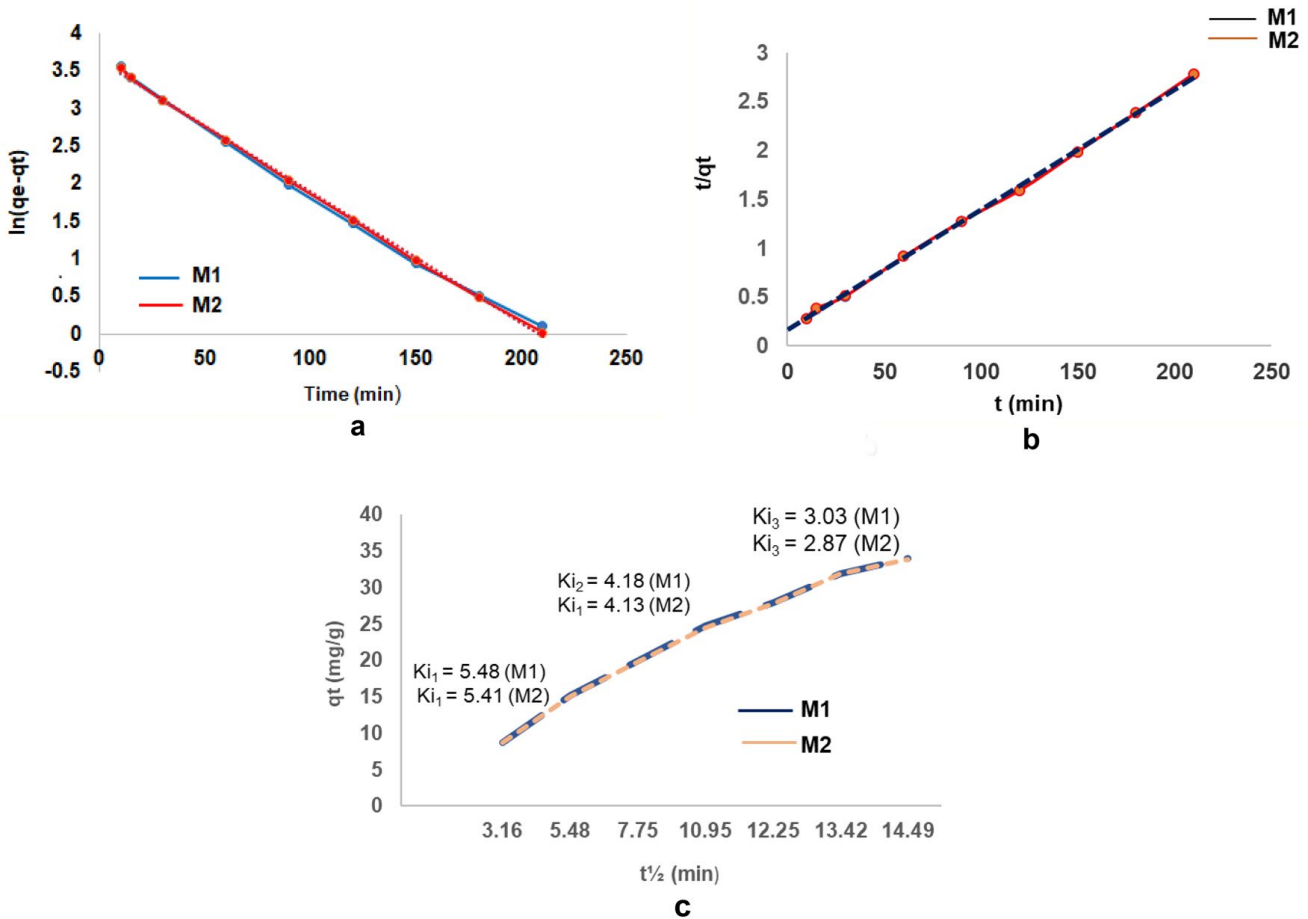
(**a**) Pseudo first-order model fitting diagram. (**b**) Pseudo second-order model fitting diagram. (**c**) Intraparticle diffusion model fitting diagram.

The kinetic models provide information on the efficiency of the adsorbent, the dynamic parameters (rate, time, etc.) of the adsorption process. The cadmium adsorption process on the prepared material was investigated employing linear and non-linear of pseudo-first-order (Eq. 6) pseudo-second-order (Eq. 7) and intraparticle diffusion models (Eq. 8) to fit the obtained experimental adsorption data. The Fig. 30a–c depicted the plots of the first-order, second-order and intraparticle diffusion models for the cadmium adsorption on nano-engineered adsorbent (EFM).

The kinetic parameters were obtained from the slope and intercept of the fitting plots of adsorption reaction models: pseudo first-order model (the correlation between log(*qe-qt*) against time), respectively the pseudo second-order model (correlation between t/qt function on time) and adsorption diffusion model: intraparticle diffusion model (the plot as function of ${t}^{1/2}$).

The results of fitting parameters on these kinetic models are presented in Table 6.

**Table 6:** Kinetic parameters for cadmium adsorption on nano-engineered adsorbent (EFM) at both molar rations (M1 and M2).

Adsorbent material	qe^exp^ (mg/g)	Pseudo first order			Pseudo second order			Intraparticle diffusion		
		q_e_^calc^	K_1_	R^2^	q_e_^calc^	K_2_	R^2^	Ki	C	R^2^
M1	75.45	75.38	0.045	0.9993	75.43	2.221	0.9998	5.487	2.5771	0.8658
M2	75.43	75.33	0.041	0.9991	75.42	2.218	0.9996	5.483	2.5129	0.8672

According to the data obtained in Table 6, the coefficients of adsorption reaction models have both values close to one, slightly differing only at the fourth decimal. It could suggest that cadmium removal is achieved through a physical and chemical adsorption process. It must be noted that were obtained higher values for the correlation coefficient (R^2^) and the calculated adsorption capacity value is very similar to those determined experimentally in the case of the pseudo-second-order kinetics model. Therefore, pseudo-second-order kinetic model was more suitable to describe the adsorption process. This indicating a chemical adsorption is assumed as the rate-limiting step for the cadmium adsorption on prepared material, involving an electron exchange between adsorbent and adsorbate (cadmium occurs with formation of strong chemical bonding)^23^.

According to the Fig. 30c the allure of intraparticle diffusion model includes three regions. The first region corresponds to a boundary diffusion (cadmium diffusion on the prepared material exterior surface). The second region is related to heavy metal intraparticle diffusion into the pores of nano-engineered adsorbent (EFM). The third region represent the cadmium adoption into the interior site of the EFM. Since, the slope of the three regions gradually decreases (Ki_1_ > Ki_2_ > Ki_3_) is assumed that boundary diffusion is the limiting region, followed by intraparticle diffusion^15,67^. The results indicate that beginning of the adsorption process cadmium ions can be quickly bound on the prepared material exterior surface. In the intraparticle diffusion process (second region) there is a gradual decrease in adsorption at the sites on the adsorbent surface (adsorption capacity reaches the maximum value). Then, cadmium adsorption takes place on the available sites inside the adsorbent, generating significant mass transfer resistance and reaching the adsorption equilibrium and the adsorption rate gradually decreases^68–70^.

The adsorption models used provide information on both the performance of the prepared material and a perspective of the adsorption mechanism.

#### Thermodynamical study

The Gibbs free energy in adsorption process was calculated according to the corresponding equation (Eq. 10). The thermodynamic parameters ΔS and ΔH were obtained from the slope and intercept of the adsorption thermodynamic curve. The obtained results are presented in next table (Table 7).

**Table 7:** Thermodynamic parameters for the cadmium adsorption on adsorbent.

T(K)	Adsorbent molar ratio					
	M1			M2		
	ΔG (kJ/mol)	ΔH (kJ/mol)	ΔS J/(mol K)	ΔG (kJ/mol)	ΔH (kJ/mol)	ΔS J/(mol K)
278.15	− 9.32	41.12	133.54	− 4.68	39.76	128.43
283.15	− 10.63			− 9.58		
298.15	− 18.88			− 17.21		
308.15	− 17.76			− 16.92		
313.15	− 16.39			− 16.34		
323.15	− 15.22			− 15.07		

From these data obtained (Table 7) can be found that the free energy variation value of the adsorption process has negative values (ΔG < 0) for both M1 and M2 indicating that adsorption is thermodynamically feasible and take place spontaneously. In the range of temperature 278.15–298.15 K, the Δ*G* values decrease with an increase of temperature. These data suggest a decrease in the feasibility of adsorption at a higher temperature. After 298.15 K a shift can be observed. The ΔG values decrease slightly with increasing temperature in the temperature range temperature: 298.15–323.15 K, indicating that the adsorption was more spontaneous at low temperature.

The calculation of the ΔH value is important because it provides information on the type of adsorption, whether it is a physical or chemical process. The ΔH value calculated in this study were 41.12 kJ/mol for M1 and 39.76 kJ/mol for M2.

The positive enthalpy values (Δ*H*) denote an endothermic adsorption process and also suggest that the type of adsorption is a physico-chemical adsorption. Likewise, the entropy charge, ΔS, positive values (133.54 J/(K mol) for M1 and 128.43 J/(K mol) indicate an affinity of this adsorbent for cadmium ions as well the fact that in the EFM adsorbent material occurs structural change^23,57,60,61,71–75^.

#### Insight on adsorption mechanism

Structural and morphological data obtained through FT-IR and SEM–EDX techniques was used to get information on cadmium adsorption mechanism on the prepared material.

**Figure 31:**
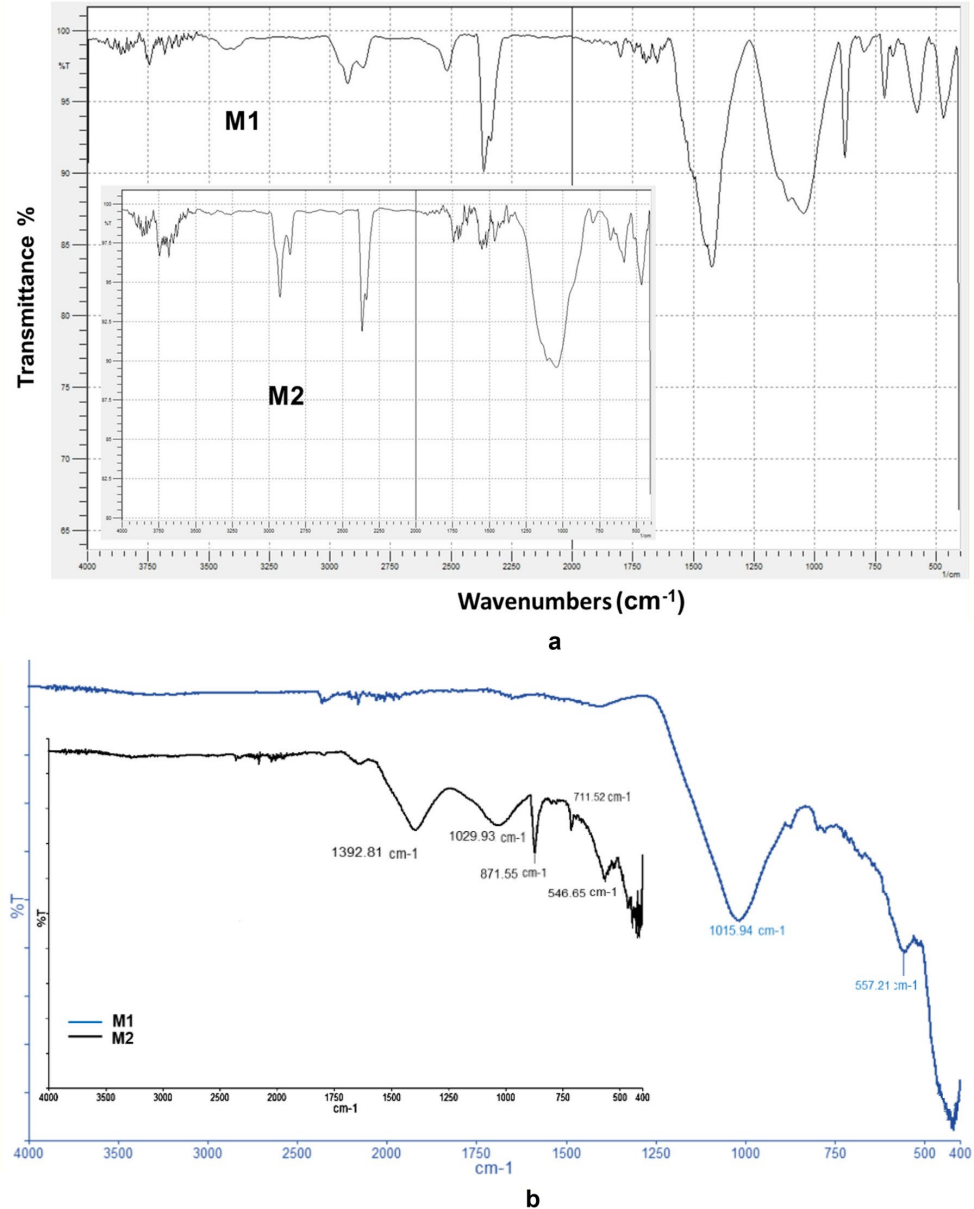
(**a**) FT-IR spectra of adsorbent (both molar ratios**: M1** and **M2**) prior adsorption. (**b**) FT-IR spectra of adsorbent (both molar ratios**: M1** and **M2**) after cadmium adsorption.

A comparative investigation on FTIR spectra of EFM was performed before (Fig. 31a) and after cadmium removal (Fig. 31b) in order to identify the modification on adsorbent functional groups vibrational bands.

Figure 31b shows significant changes in the intensity of some vibrational peaks, the displacement of others at other wavelengths and new absorption bands identified after adsorption of cadmium on EFM. Also, new adsorption bands and substantial changes regarding the intensity and displacement of functional groups peaks from M1 and M2 after the cadmium adsorption can be observed in Fig. 31b. Thus, the significant attenuation of the O–H peak at 3740 cm^−1^ can be attributed to the fact that this functional group is involved in the adsorption process^23,68^.

In addition, the cadmium adsorption on EFM determined a significant attenuation of the intensity of the following adsorption bands: 1423 cm^−1^ (attributed to C–O stretching vibration), at 1798 and 2515 cm^−1^ (associated with O–C–O), at 2875 respectively at 2981 cm^−1^(CH– symmetric and asymmetric stretching vibration). These differences in the band intensity can be attributed to the interaction of cadmium ions with functional groups of EFM^76^.

After the cadmium adsorption on M1 and M2It can also be seen that the absorption bands at about: 589 cm^−1^ (associated with Fe–O stretch vibration), 670 cm^−1^ (attributed to the Al–O–Al bending), 875 cm^−1^ (attributed to C–O stretching vibration) and 1100 cm^−1^(associated with X–O (X = Al, Si)) have been shifted.

Peak identified at 1392.81 cm^−1^ can be attributed to the nitrate stretching vibration from the heavy metal source (cadmium nitrate)^77,78^.

Also, the appearance of new absorption bands in the 400–500 cm^−1^ region can be attributed to the Cd–O bond vibrational peaks^77^. According to the FTIR results can assume that adsorption of cadmium on EFM takes place through chemical bonds^76,77^.

To confirm the results of FTIR spectroscopy, the morphological modification of the EFM after cadmium adsorption was investigated by the SEM_EDX technique.

**Figure 32:**
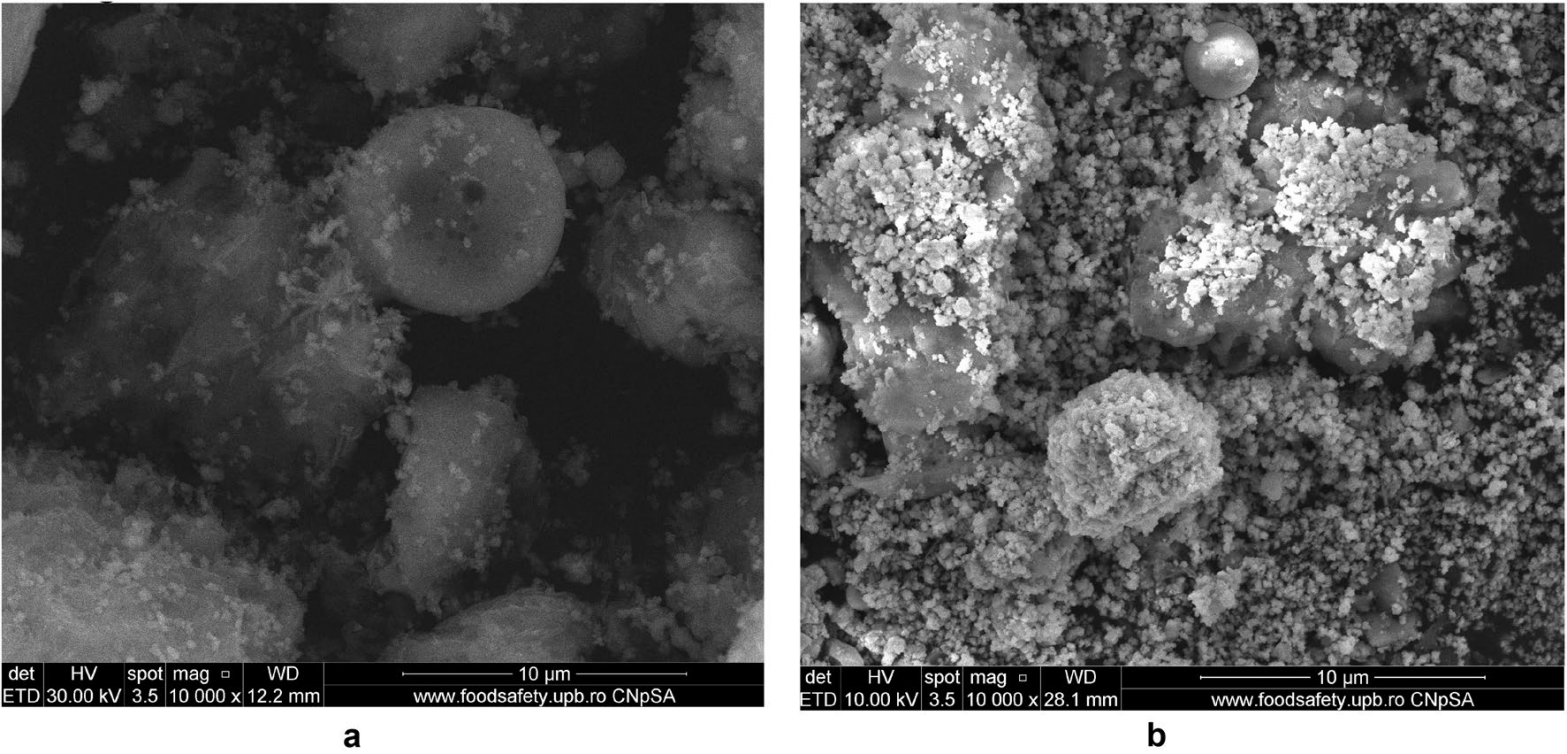
(**a**) Two-dimensional image of **M1** particle obtained by the SEM technique (magnitude 10 µm) before adsorption. (**b**) Two-dimensional image of **M1** particle obtained by the SEM technique (magnitude 10 µm) after adsorption.

**Figure 33:**
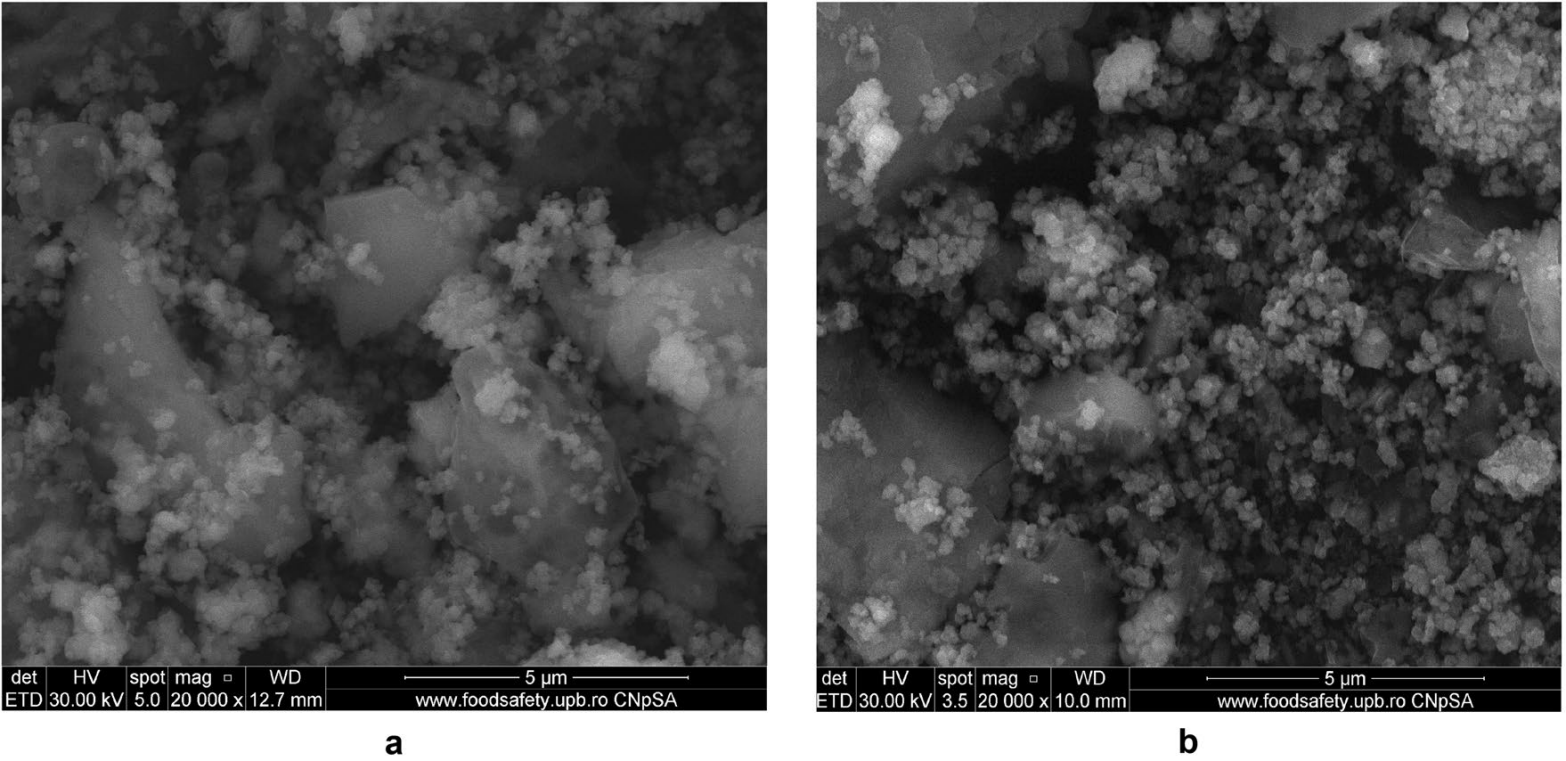
(**a**) Two-dimensional image of M2 particle obtained by the SEM technique (magnitude 5 µm) before adsorption. (**b**) Two-dimensional image of M2 particle obtained by the SEM technique (magnitude 5 µm) after adsorption.

The SEM micrograph of M1 and M2 before and after the cadmium adsorption are presented in the Figs. 32 and 33.

The SEM micrograph of M1 and M2 after adsorption (Figs. 32b, 33b) indicate the presence of the presence of numerous particles of irregular shapes attributed to heavy metal. Since both SEM of M1 and M2 after adsorption shows a higher accumulation of particles compared with SEM images before (Figures), which suggests that adsorption takes place in the inner pores of the prepared material and thus decreasing the porosity of the adsorbent. This fact indicates that both M1 and M2 show morphological changes after cadmium adsorption^76,79^.

**Figure 34:**
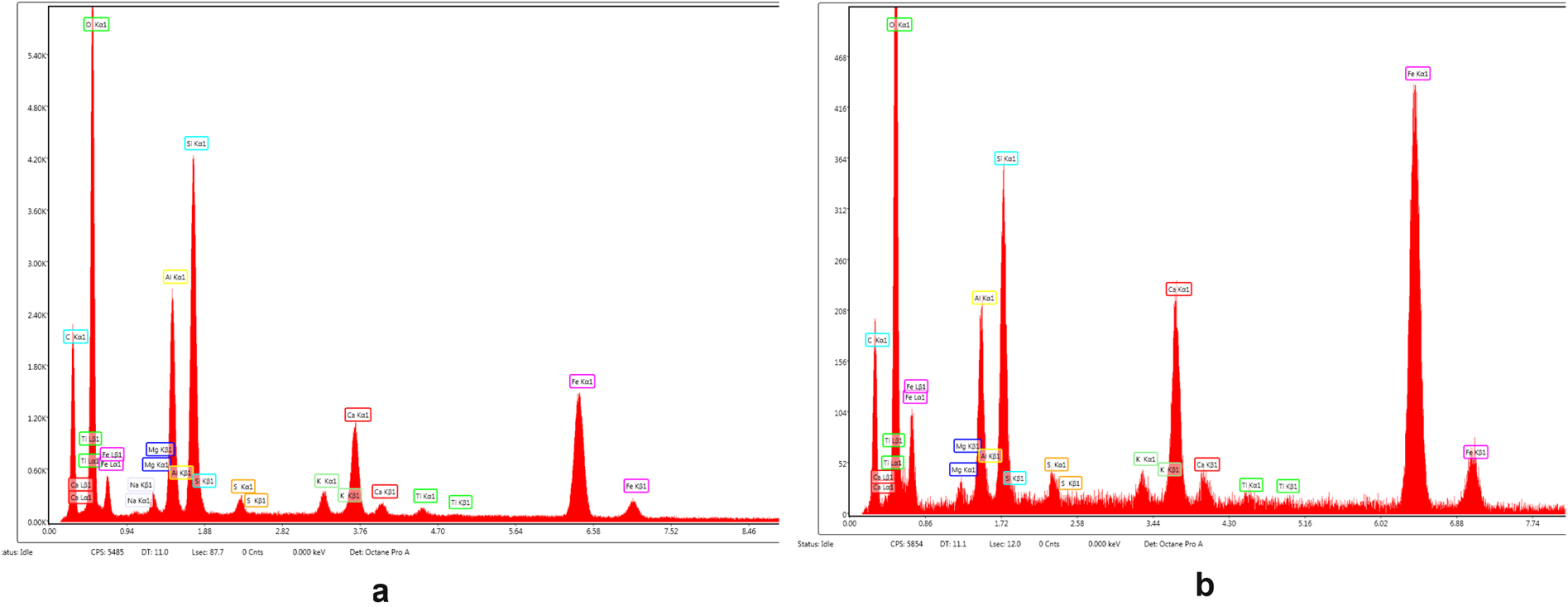
(**a**) EDX spectra of **M1**prior adsorption. (**b**) EDX spectra of **M2** prior adsorption.

**Figure 35:**
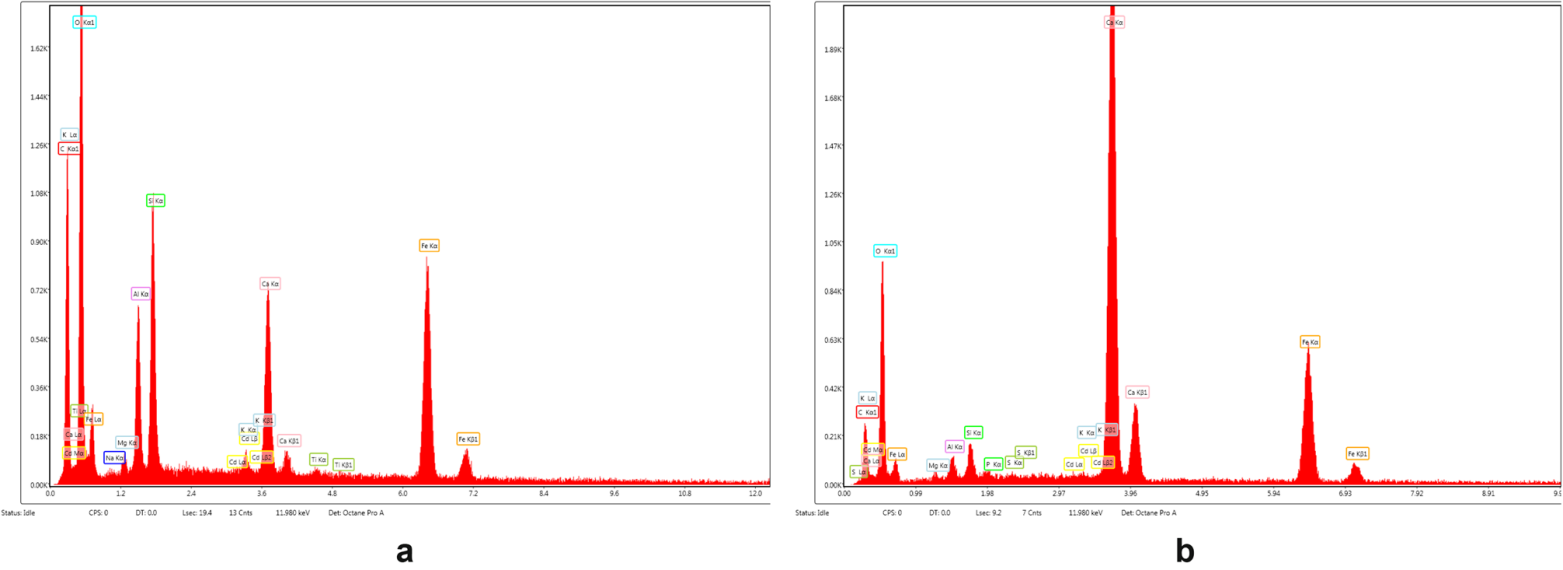
(**a**) EDX spectra of M1 after adsorption. (**b**) EDX spectra of M2 after adsorption.

The EDX spectra of M1 and M2 prior and after adsorption are presented in the Figs. 34 and 35.

The intensity of M1 and M2 peaks corresponding to their elemental composition differs due to the different molar ratios of the two raw materials (ash and eggshell) that are part of the adsorbent component.

The comparative analysis of the Figs. 34 and 35 shows that in the EDX spectra of M1 and M2 the peak of the heavy metal appears only after adsorption (Figs. 34b, 35b).

According to these results the adsorption of cadmium takes place into the EFM^76,79,80^.

#### Desorption study

A sustainable and efficient adsorbent means very high efficiency of pollutant removal and the reused possibility^15,59^.

In this study, the desorption of the adsorbed cadmium ions on the EFM was investigated using acid (nitric acid and hydrochloric acid) and base (NaOH) solutions. The desorption yield was determined using the equation (Eq. 13).

**Figure 36:**
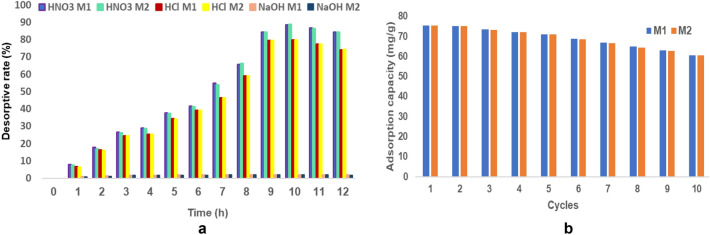
(**a**) The relationship between the desorption rate and time. (**b**) Reuse of EFM.

Figure 36a shows the relationship between the desorption rate obtained for the desorption agents used as a function of time. It can be observed as the desorption rate, for all desorption agents used, increases proportionally with time between 0 and 10 h, when it reaches a maximum value, after which there is a slight decrease^57^.

According to Fig. 36a, nitric acid was the best desorption agent (a maximum desorption rate of 88.56% for M1 and 88.82% for M2). This fact can be justified that, in an acidic environment, there is a massive number of protons competing with adsorbed cadmium ions. The increase in electrostatic repulsion forces between cadmium ions leads to the replacement of cadmium with protons in the active sites of the adsorbent^15,59^.

Instead, in an alkaline environment occurs the cadmium precipitation, although, in the presence of NaOH, are desorbed a large number of cadmium ions from the surface of the prepared material^59^.

#### Adsorbent regeneration

The key feature of a cheap and high-performance adsorbent is the possibility of multiple reuses. And the desorption of the pollutant from the adsorbent must be done by a simple and efficient method^68^.

Ten cycles of adsorption–desorption experiments were conducted to examine the cadmium adsorption capacity of the EFM. Figure 36b depicted the variation of the adsorption capacity of cadmium depending on the number of adsorption/desorption cycles. It was found that the adsorption capacity decreased by 20% (both for M1 and M2) after ten cycles. After only five cycles, the adsorption capacity decreased by only 6% for M1 and M2. These results suggest that the performance and regenerative capacity of EFM are good^23,61^.

#### ANOVA test

Table 8 present the results of one-way analysis of variance (ANOVA) without replication.

**Table 8:** ANOVA single factor- statistical parameters.

Source of variation	Sum of square (SS)	Degree of Freedom (df)	Mean squares (MS)	*F*	*P value*	*F crit*
Between groups	1135.185	1	1135.185	1.149592	0.30177	4.60011
Within groups	13,824.54	14	987.4672	–	–	–
Total	14,959.73	15	–	–	–	–

The results obtained (F = 1.149592 and *p* = 0.30177) indicate that variation within the samples (F) is a value for F ~ 1, and the *p* value is > α lower than 0.05 (α = 0.05), which suggests that between the M1 and M2 there are not statistically significant differences.

## Conclusions

This study described the cadmium removal from an aqueous solution using a newly engineered nano-adsorbent (EFM) from two different wastes (eggshell and fly ash). The adsorption and physico-chemical properties of this new adsorbent were detailed studied. The mechanism of functionalization was the simultaneous loading of each waste (eggshell and fly ash) with magnetite nanoparticles and was confirmed by SEM, XRD, and FTIR.

In addition, SEM and BET analysis support the claim that the double functionalization of the eggshell with ash particles functionalized with magnetite was achieved simultaneously with the loading of the pores of the eggshell surface with the magnetite particles. Therefore, there was a substantial increase in the surface area and the number of active adsorption centers, which allowed to obtain a very high cadmium removal efficiency (99.9%). The room temperature and pH 6.5 are favourable for the adsorption process. Results from the adsorption isotherms study indicated that adsorption for cadmium ions was a multi-molecular layer adsorption process that occurs on the investigated new magnetic nano-sized engineered adsorbent heterogeneous surfaces. The adsorption kinetics results suggest a chemical mechanism described by pseudo second-order model. FTIR and SEM–EDX studies suggest that metal adsorption on the new magnetic nano-sized engineered adsorbent is done through chemical bonds.

Desorption and regeneration study shows that nitric acid can be used for the cadmium desorption (a maximum desorption rate of 88.56% for M1 and 88.82% for M2) and also for the adsorbent regeneration.

This study demonstrates the advantages of this newly engineered adsorbent: (1) simple preparation method; (2) environmental-friendly; (3) reusable; (4) highly efficient at mild experimental condition (25 °C pH 6.5); (5) stable and (6) simple, easy method to reuse a mixt of waste within the circular economy model for heavy metal wastewater treatment.
